# Sustainable Development of High‐performance Poly(ester‐imine) Biobased Thermosets

**DOI:** 10.1002/advs.202503483

**Published:** 2025-04-25

**Authors:** Roxana Dinu, Sandu Cibotaru, David D. Swanson, Alice Mija

**Affiliations:** ^1^ Université Côte d'Azur ICN France; ^2^ Air Force Office of Scientific Research (AFOSR) European Office of Aerospace Research and Development (EOARD) 86 Blenheim Crescent, Ruislip HA4 7HD UK

**Keywords:** biobased thermosets, high‐performance, novel poly(ester‐imine), Schiff bases

## Abstract

The massive production of materials based on petroleum derivatives and often toxic compounds generates significant environmental pollution. In this context, the development of environmentally friendly alternatives becomes crucial. This study investigates the synthesis of eco‐friendly and high‐performance poly(ester‐imine) thermosets by sustainable approaches. Starting with vanillin or syringaldehyde, the synthesis involves the introduction of imine linkages within the epoxy monomer's structure. A new strategy is used through the cross–linking of the designed epoxy monomers using anhydrides, without initiators. This approach conducts to novel poly(ester‐imine) thermosets with high performances that exceed those reported in previous studies. The thermo‐mechanical investigations show that the novel‐designed thermosets have performant properties with storage moduli at room temperature ranging between 0.3‒1.74 GPa and glass transition values between 90 and 170 °C. The Limit Oxygen Index (LOI) in the range of 24‒36%, confirms the excellent flame‐retardant properties without the addition of supplementary additives. Their low‐density values (1.05‒1.36 g cm^−3^) and the very low WA% ≈0.09–1.15 % after 24 h make them well‐suited for a range of uses where weight and hydrophobicity considerations are critical. The obtained results place these poly(ester‐imine) materials as promising sustainable candidates for a wide range of high‐end applications.

## Graphical abstract

Schiff‐based epoxy monomers derived from renewable natural resources are used to design robust structures by cross–linking with bio‐anhydrides. The resulting eco‐friendly, high‐performance thermosets feature two cleavable bonds, offering versatility for various applications. These materials exhibit properties suitable for a wide range of needs, from flexible to rigid structures, ensuring adaptability in demanding environments.

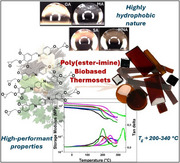

## 1 Introduction

A wide range of environmental challenges confront us, with significant issues including global warming, intensified by emissions from fossil fuels, and the widespread problem of plastic pollution.^[^ ^1^ ^]^ Despite their essential roles in numerous industries and daily life, both fossil fuels and plastics significantly contribute to roles in environmental degradation. Notably, the production of plastics is closely linked to the extraction and processing of fossil fuels, thereby intensifying carbon emissions throughout their entire lifecycle. This interconnection highlights the profound impact of these materials on the environment and underlines the urgent need for sustainable alternatives and practices.^[^ ^2^ ^]^

Epoxy thermosets are renowned for their exceptional mechanical strength, chemical resistance, thermal stability, and electrical insulation properties.^[^ ^3^ ^]^ They currently account for ≈20% of global plastic production,^[^ ^4^ ^]^ and are extensively applied in critical industries such as aerospace,^[^ ^5^ ^]^ electronics,^[^ ^6^ ^]^ automotive,^[^ ^7^ ^]^ and naval sectors.^[^ ^8^ ^]^ However, the widespread use of bisphenol A (BPA) as a key precursor in traditional epoxy formulations raises significant environmental and health concerns.^[^ ^9^ ^]^ Furthermore, traditional epoxy thermosets made from BPA lack recyclability, thus limiting their recovery and reuse to <10% after initial use.^[^ ^10^ ^]^ To address these environmental and health challenges, there is an increasing focus on developing sustainable alternatives in epoxy resin chemistry. Researchers are investigating biobased sources, such as lignin‐derived compounds and renewable plant‐based materials, to replace BPA.^[^ ^11^ ^]^

The scientific community's increased focus on lignin, a major component of pulp and paper industry waste alongside cellulose. Lignin remains largely underutilized, often ending up as landfill waste, posing environmental challenges due to its inherent resistance to microbial degradation and potential for spontaneous combustion.^[^ ^12^ ^]^ One promising area of research involves converting lignin into value‐added products, such as phenolic compounds, biobased polymers, and specialty chemicals.^[^ ^13^, ^14^ ^]^ This transformation typically involves depolymerization processes that break down lignin into smaller molecular units, including aromatic compounds like vanillin and syringaldehyde. Vanillin (VAN), a key component of natural vanilla extract known for its aroma and flavoring properties, can be derived from lignin through various methods, including acid hydrolysis and oxidative processes. Industrial production of vanillin from lignin involves the controlled depolymerization of lignin under specific conditions to yield high‐purity vanillin suitable for commercial applications.^[^ ^15^, ^16^, ^17^ ^]^ Similarly, syringaldehyde (SYR), another aromatic aldehyde with distinctive fragrance and chemical versatility, can be synthesized from lignin‐derived precursors using oxidative processes like the Duff reaction. This method involves the controlled oxidation of lignin‐derived phenolic compounds to convert them into syringaldehyde, which is used in flavors, fragrances, and pharmaceuticals.^[^ ^18^ ^]^ These efforts not only address environmental concerns but also support the development of renewable resources for a sustainable future.

Schiff bases are typically synthesized through reactions between aldehydes and amines, where the selection of aromatic amines with azomethine bonds directly linked to the aryl ring plays a pivotal role.^[^ ^19^ ^]^ This strategic choice enhances the thermal stability and mechanical properties of the designed structures. Schiff‐based epoxy thermosets are commonly cured using amines as cross–linking agents, resulting in materials known for their robust thermal stability and mechanical properties.^[^ ^20^, ^21^, ^22^, ^23^, ^24^, ^25^, ^26^ ^]^ In recent developments, Schiff‐based epoxy resins have emerged as promising alternatives to conventional BPA‐based epoxies, aiming to address sustainability and recyclability requirements. For example, Li et al.^[^ ^27^ ^]^ reported the synthesis of a Schiff‐based thermoset with a notably high bio‐based content (up to 96%), derived from vanillin and modified guaiacol. The resulting thermoset exhibited good thermo‐mechanical properties, including a glass transition (*T*_g_) value of ≈220 °C and a storage modulus (*E*') of 3602 MPa. Similarly, Yang et al.^[^ ^28^ ^]^ designed a novel di‐Schiff‐based epoxy thermoset using vanillin as the bio‐based precursor and 9,9‐bis(4‐aminophenyl)fluorene cured with DDM hardener. The obtained thermoset demonstrated promising characteristics such as degradability, intrinsic flame retardancy, and low water absorption, positioning it as a sustainable material. In an effort to further enhance the bio‐based content and multifunctionality of epoxy thermosets, Nabipour et al.^[^ ^29^ ^]^ developed an epoxy‐tri‐vanillin Schiff‐base compound using triamino guanidine. The obtained thermosets exhibited *T*_g_ values of ≈224 °C and a storage modulus at 25 °C of ≈3383 MPa. Despite these advancements, the curing agents used in these systems remain petroleum‐derived, specifically the 4,4′‐diamino diphenylmethane (DDM). To address this limitation, Nabipour et al.^[^ ^30^ ^]^ proposed a fully bio‐based thermoset using a syringaldehyde‐based Schiff‐base epoxy monomer paired with a furan‐based diamine curing agent (DIFFA). While this bio‐based thermoset exhibited good thermo‐mechanical properties, including a *T*_g_ of ≈204 °C, *E*’ at 25 °C of ≈2188 MPa, and a limiting oxygen index (*LOI*) of 40%, challenges such as the complex synthesis pathway and low yield of the synthesized diamine curing agent were identified.

To address the issue of petroleum‐based or toxic curing agents in Schiff‐based epoxy thermosets, an alternative strategy involves replacing diamines with anhydrides, offering a more sustainable approach while maintaining the required material properties. Considering the aforementioned aspects, the goal of this study is to develop Schiff‐based epoxy thermosets by employing biobased compounds to create azomethine‐containing monomers and thermosets. The biobased compounds selected for epoxy monomer synthesis were vanillin (VAN) and syringaldehyde (SYR). These phenolic aldehydes, of natural origin, are both derived from lignin. Using these compounds, four epoxy monomers were synthesized as previously reported,^[^ ^31^ ^]^ starting with the creation of a biphenolic structure containing one or two azomethine bonds, which were then glycidylated to introduce the epoxy functionality. For thermosets development, a new and sustainable approach was applied to advance beyond previously reported Schiff‐based epoxy thermosets. Therefore, the synthesized vanillin and syringaldehyde‐based epoxy Schiff‐base monomers were cross–linked with various biobased anhydrides. Maleic anhydride (MA) can be produced using renewable resources, such as bio‐based butane derived from biomass or various furan compounds.^[^ ^32^, ^33^, ^34^, ^35^ ^]^ For instance, furfural, obtained from lignocellulosic agricultural waste, can be converted into maleic anhydride through a series of oxidation and subsequent reactions.^[^ ^36^ ^]^ In addition to maleic anhydride, furfural can also be processed to form *cis*‐1,2,3,6‐tetrahydrophthalic anhydride (THPA) through specific chemical transformations.^[^ ^37^ ^]^ Similarly, succinic anhydride (SA) is synthesized by dehydrating bio‐based succinic acid. These anhydrides, including maleic and succinic anhydride, can be produced via sustainable processes utilizing biomass‐derived feedstocks like furan derivatives or organic acids. While phthalic anhydride (PA) is typically derived from petroleum sources,^[^ ^38^ ^]^ significant advances have been made in developing renewable production methods, positioning these phthalic anhydride derivatives as viable bio‐based alternatives for the future.^[^ ^36^, ^39^, ^40^ ^]^

The main objective of this study was to obtain sustainable, eco‐friendly, and high‐performance poly(ester‐imine) thermosets with tunable properties, making them suitable for a wide range of industrial applications. The physicochemical and thermomechanical properties of the designed thermosets were investigated using various techniques, including Differential Scanning Calorimetry (DSC), Attenuated Total Reflection Fourier Transform Infrared Spectroscopy (ATR‐FTIR), Thermogravimetric Analysis (TGA), Dynamic Mechanical Analysis (DMA), Shore Hardness tests, Water Absorption (*WA*, %) tests, and Gel Content (*GC*, %) measurements. Through these efforts, this study aims to advance the development of sustainable materials by leveraging biobased resources and innovative chemical strategies.

## 2 Results and Discussions

### 2.1 Curing Behavior of Schiff‐base Epoxy Monomers With Anhydrides

**Figure 1:**
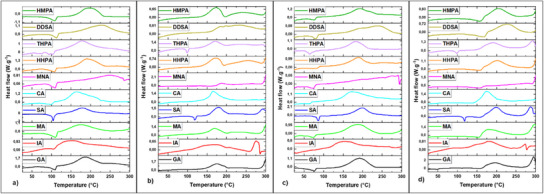
DSC thermograms registered during heating at 10 °C per min the mixtures with various anhydrides of a) 4‐((oxiran‐2‐yl)methoxy)‐N‐(4‐((oxiran‐2‐yl)methoxy)‐3‐methoxy benzylidene) benzene amine (EP‐VAN‐AP), b) N1, N4‐bis (4‐((oxiran‐2‐yl)methoxy)‐3‐methoxy benzylidene) benzene‐1,4‐diamine (EP‐VAN‐DiAP), c) 4‐((oxiran‐2‐yl)methoxy)‐N‐(4‐((oxiran‐2‐yl)methoxy)‐3,5‐dimethoxy benzylidene) benzene amine (EP‐SYR‐AP), and d) N1,N4‐bis(4‐((oxiran‐2‐yl)methoxy)‐3,5‐dimethoxybenzylidene)benzene‐1,4‐diamine (EP‐SYR‐DiAP).

The non‐isothermal curing behavior (**Figure** **1**) of the Schiff‐based epoxy monomers with the selected anhydrides was studied using Differential Scanning Calorimetry (DSC). This study aims to investigate the thermal reactivity of these systems and to determine the optimal conditions for their curing process.

The specific curing temperatures, including the start and end of the reaction, the maximum peak temperature of the reaction (*T*_peak_), and the total heat release during the complete curing process (Δ*H*) for each system, are summarized in **Table** **1**.

**Table 1:** DSC data for the copolymerization of Schiff‐based epoxy monomers with various anhydrides.

System	EP‐VAN‐AP			EP‐VAN‐DiAP			EP‐SYR‐AP			EP‐SYR‐DiAP		
	*T*_peak_ [°C]	Reaction Interval [°C]	Δ*H*[J g^−1^]	T_peak_[°C]	Reaction Interval [°C]	Δ*H* [J g^−1^]	*T*_peak_ [°C]	Reaction Interval [°C]	Δ*H* [J g^−1^]	*T*_peak_[°C]	Reaction Interval[°C]	Δ*H* [J g^−1^]
GA	186 ± 0.7	150‐234 ± 1.4	332 ± 9	175 ± 0.6	96‐206 ± 1.6	194 ± 8	192 ± 0.5	132‐238 ± 1.7	373 ± 9	195 ± 0.5	167‐263 ± 1.5	277 ± 10
IA	152 ± 0.8	114‐230 ± 1.7	172 ± 7	168 ± 0.7	99‐202 ± 1.5	101 ± 6	146 ± 0.8	112‐264 ± 1.6	223 ± 8	179 ± 0.6	113‐237 ± 1.7	239 ± 8
MA	174 ± 0.65	128‐251 ± 1.6	280 ± 8	169 ± 0.7	102‐202 ± 1.4	167 ± 8	189 ± 0.7	117‐250 ± 1.4	383 ± 8	178 ± 0.4	160‐256 ± 1.6	278 ± 8
SA	179 ± 0.9	133‐237 ± 1.3	320 ± 10	179 ± 0.8	131‐210 ± 1.5	220 ± 9	199 ± 0.7	138‐257 ± 1.4	355 ± 10	199 ± 0.7	168‐253 ± 1.7	326 ± 10
CA	164 ± 0.85	163‐223 ± 1.8	351 ± 6	165 ± 0.7	106‐238 ± 1.8	317 ± 10	172 ± 0.9	121‐249 ± 1.5	279 ± 8	175 ± 0.7	146‐238 ± 1.4	355 ± 9
MNA	246 ± 0.75	178‐280 ± 2	100 ± 8	187 ± 0.9 263 ± 0.7	165‐224 ± 1.7 226‐289 ± 1.4	62 ± 5 19 ± 3	196 ± 0.9	140‐223 ± 1.9	15 ± 6	276 ± 0.8	210‐290 ± 1.6	30 ± 7
HHPA	190 ± 1	144‐244 ± 1.7	339 ± 7	170 ± 0.8 255 ± 0.7	107‐194 ± 1.7 210‐290 ± 1.8	132 ± 6 66 ± 4	189 ± 0.8	114‐227 ± 1.8	182 ± 7	201 ± 0.7	166‐281 ± 1.9	270 ± 8
THPA	179 ± 0.95	131‐244 ± 1.5	275 ± 7	170 ± 0.6 243 ± 0.7	119‐199 ± 1.7 201‐273 ± 1.7	144 ± 5 40 ± 4	182 ± 0.7	110‐230 ± 1.7	279 ± 9	190 ± 0.9	149‐246 ± 1.8	248 ± 7
DDSA	230 ± 0.85	160‐281 ± 1.9	324 ± 9	198 ± 0.9	141‐291 ± 1.9	180 ± 7	240 ± 0.6	132‐292 ± 1.7	280 ± 9	225 ± 0.5	179‐285 ± 2	200 ± 5
HMPA	197 ± 0.90	139‐241 ± 1.7	330 ± 8	173 ± 0.8 239 ± 0.9	109‐196 ± 1.8 197‐291 ± 1.6	144 ± 7 77 ± 4	193 ± 0.7	117‐246 ± 1.6	234 ± 6	203 ± 0.7	169‐260 ± 1.8	285 ± 8

The DSC thermograms obtained during heating at 10 °C per min for the epoxy monomers EP‐VAN‐AP and EP‐SYR‐AP show an endothermic peak associated with the melting of these compounds (Figure S1a, Supporting Information), occurring around *T*_m_ = 123 °C for EP‐VAN‐AP and *T*_m_ = 95 °C for EP‐SYR‐AP. As seen in Figure 1, the cross–linking reactions of EP‐VAN‐AP and EP‐SYR‐AP with anhydrides are characterized by a main exothermic peak. In the case of systems with EP‐VAN‐DiAP and EP‐SYR‐DiAP, the melting of these monomers occurs at higher temperatures: *T*_m_ = 205 °C for EP‐VAN‐DiAP and *T*_m_ = 180 °C for EP‐SYR‐DiAP. This is attributed to their higher molar mass: *M*_n_ (EP‐VAN‐DiAP) = 488 g mol^−1^ and M_n_ (EP‐SYR‐DiAP) = 548 g mol^−1^ compared to the lower molar mass of EP‐VAN‐AP *M*_n_ = 355 g mol^−1^ and EP‐SYR‐AP *M*_n_ = 385 g mol^−1^. As shown in Figure 1, the DSC thermograms for EP‐VAN‐DiAP and EP‐SYR‐DiAP curing with anhydrides display an initial exothermic peak, indicative of the epoxy‐anhydride copolymerization followed by a second exothermic peak occurring at temperatures above 280 °C, which is likely due to some secondary reactions, as self‐polymerizations or etherifications, but also to a start in thermal decompositions. As previously reported,^[^ ^31^ ^]^ the self‐homopolymerization reactions of these Schiff‐based epoxy monomers occurred at *T* ≈ 225–250 °C.

Regarding the reaction enthalpy, it is observed that the curing systems with EP‐VAN‐AP and EP‐SYR‐AP exhibit higher Δ*H* values compared to those involving EP‐VAN‐DiAP and EP‐SYR‐DiAP. This difference can be attributed to the higher melting temperatures of EP‐VAN‐DiAP and EP‐SYR‐DiAP monomers, which can occur simultaneously with the curing, so the endothermal melting is hindered by the exotherm of the reaction (Figure S1, Supporting Information). Therefore, the melting of EP‐VAN‐DiAP and EP‐SYR‐DiAP occurring concomitantly with exothermal curing reduces the total heat release (Δ*H*) of the cross–linking process.^[^ ^41^, ^42^, ^43^, ^44^ ^]^

For the EP‐VAN‐AP curing systems, an exothermic peak begins in the range *T*_onset_ = 110‒200 °C and reaches a maximum around *T*_peak_ = 152‒230 °C, followed by a quasi‐linear response around *T*_end_ = 220‒280 °C. The reaction enthalpy for these systems ranges from ≈172 to 340 J g^−1^, depending on the anhydride. In the EP‐VAN‐DiAP system, the reaction range start at *T*_onset_ = 90‒170 °C, and finish at *T*_end_ = 190‒290 °C, with a maximum reactivity temperature at *T*_peak_ = 165‒255 °C. The reaction enthalpy in these systems ranges from 62 to 220 J g^−1^, except for the system with CA, which exhibits a higher enthalpy of ≈320 J g^−1^. Furthermore, EP‐VAN‐DiAP systems display a secondary reaction peak between 200 °C and 290 °C, with an enthalpy of 19 to 77 J g^−1^. This secondary peak may involve additional secondary reactions as previously discussed (homopolymerisations, thermal degradations). For the EP‐SYR‐AP cross–linking systems, the Δ*H* varies from 180 to 380 J g^−1^, with the maximum reactivity occurring at *T*_peak_ = 145‒240 °C (*T*_onset_ = 110‒140 to *T*_end_ = 230‒290 °C). In contrast, the EP‐SYR‐DiAP formulations exhibit an exothermic peak with the enthalpy Δ*H* ranging from 200 to 350 J g^−1^, and a maximum reactivity at *T*_peak_ = 175‒225 °C (*T*_onset_ = 110‒180 to *T*_end_ = 230‒290 °C). In some EP‐SYR‐DiAP formulations, also a second exothermic peak appears, occurring between 260‐290 °C. These variations in reaction ranges and enthalpies underscore the influence of the molecular structure of Schiff‐based monomers and the choice of anhydride on the curing behavior of these systems.

**Figure 2:**
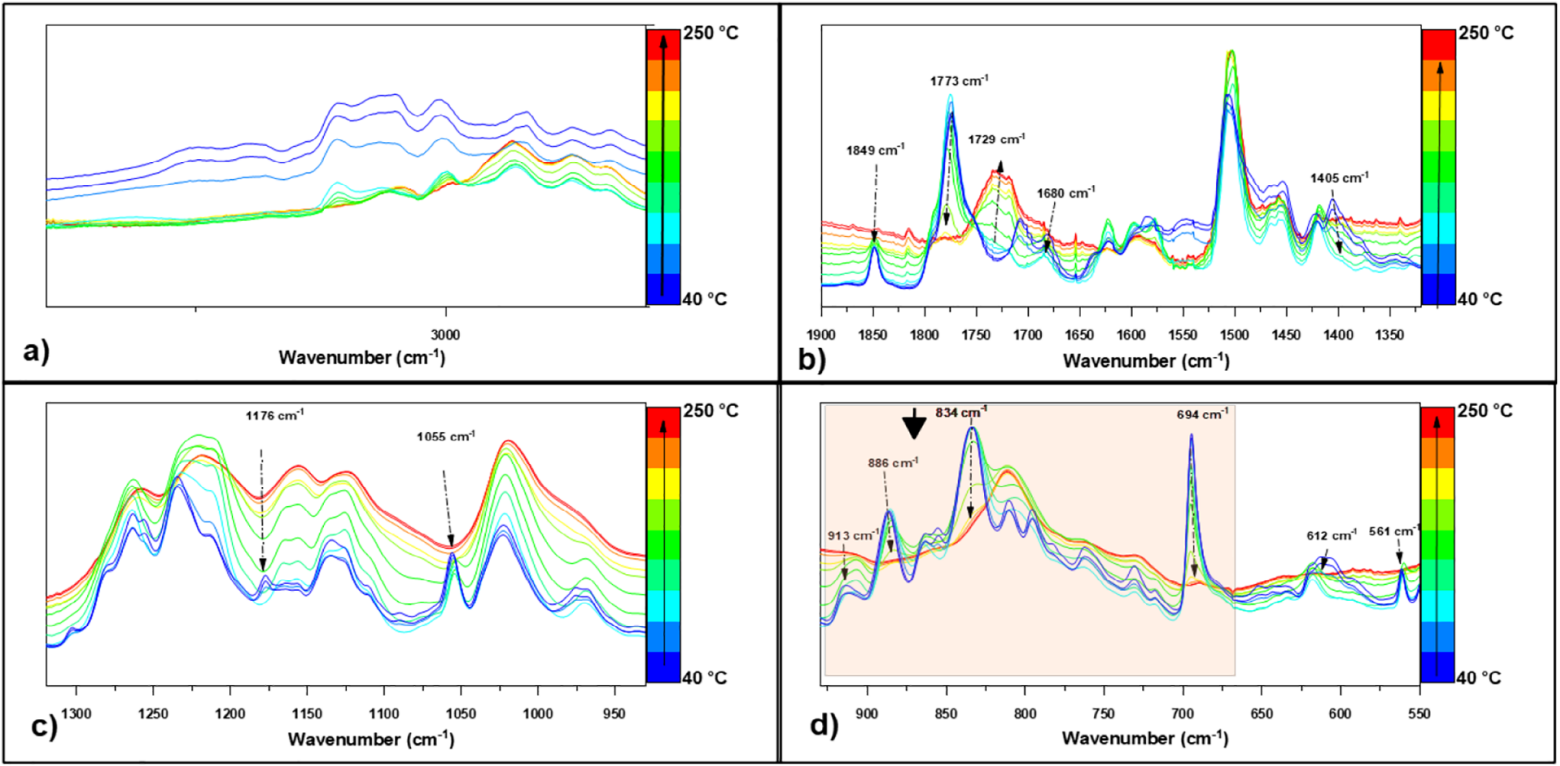
(a–d)Temperature assisted FTIR spectra of EP‐SYR‐AP/MA system.

The chemical evolution during the curing of the Schiff‐based epoxies with anhydrides was investigated by using FT‐IR spectroscopy (**Figure** **2**; Figures S2 and S3a–d, Supporting Information). In the initial stage of the heating process, the characteristic absorption bands for both anhydrides and epoxy monomers are clearly defined. For example, the bands at 1850‒1848 and 1774‒1772 cm^−1^ correspond to the symmetric and asymmetric stretching vibration of the C═O bond in the MA anhydride, respectively, while those in the region of 1690‒1680 cm^−1^ are associated with the C═C stretching vibration. The imine characteristic band from the Schiff‐based epoxy monomer appears ≈1624‒1618 cm^−1^ (Figure 2, Figures S2 and S3a–d).

Depending on the formulation, the maximum absorption band can vary slightly. During the heating process (Figure 2, Figures S2 and S3a–d, Supporting Information) a decrease in the anhydride bands is observed, along with the appearance of a new band ≈1725 cm^−1^, indicating the opening of the anhydride ring and the formation of a new ester bond. The ester bond appears to be broad due to the high number of repeating ester units in the polymer chain. Similarly, the bands at 1050 and 698 cm^−1^, which are characteristic of C─O─C stretching vibration and disubstituted C═C bending vibrations, respectively, decreased during the heating, confirming the anhydride ring opening. The band at 1121‒1156 cm^−1^, which appears as a broad absorption band after temperature treatment, suggests the formation of new C─O ester bonds. Additionally, the epoxy ring absorptions at ≈913‒904, 886, and 834 cm^−1^ also decrease indicating their involvement in the anionic copolymerization with the anhydride. The region between 1075–1025 cm^−1^ was monitored (the red spot in Figure 2; Figures S2 and S3c, Supporting Information) as an indicator of ether group appearance.

**Scheme 1:**
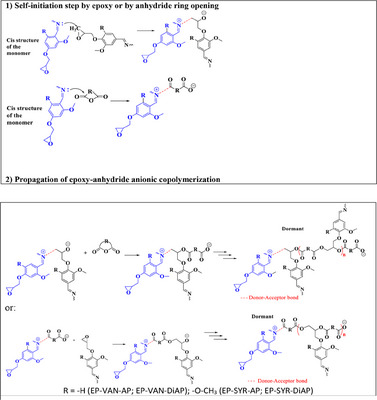
Schiff‐based epoxy monomer copolymerization mechanism with anhydride by self‐initiation.

Given that the copolymerization reaction between Schiff‐based epoxy monomers and anhydrides occurred without an initiator, it may be the result of a self‐initiation process. In this case, the Schiff‐based epoxy monomers contain an imine bond within a highly conjugated structure. Due to this, the nitrogen atom can readily donate its electron pair, forming a cation that is stabilized by the conjugation effect.^[^ ^45^, ^46^ ^]^ The donated electrons can be transferred to the epoxy ring or to the anhydride, in both cases resulting in the formation of an oxyanion structure capable of initiating the anionic copolymerization^[^ ^47^ ^]^ (**Scheme** **1**). In this manner, copolymerization occurs, keeping the anionic charge in a dormant living state.

The thermosets developed in this study contain repeated imine units within their monomer structure. However, the polymer network formed after the epoxy‐anhydride curing process is distinctly a polyester, even if some homopolymerization reactions can occur at high temperatures leading to some polyether structures. Therefore, these materials are referred to as poly(ester imine) thermosets, reflecting both the polyester nature of the cured polymer network and the presence of imine functional groups.

Following the DSC analyses, a standardized cross–linking protocol was established for all formulations, with the materials subsequently developed in an oven for various tests. It is mentioned that formulations involving IA and CA anhydrides, as well as EP‐SYR‐DiAP formulations with GA, MA, and SA, resulted in porous materials. Due to their porous nature, these materials were considered unsuitable for structural thermoset applications. Therefore, they were discarded from further investigation in this context but will be considered for other applications where their properties might prove advantageous.

To ensure the validity of the manufacturing cross–linking program, the gel fraction of the systems was measured using the solvent method. The results, presented in **Table** **2**, indicate that the gel content of the tested thermosets exceeds 99%, with values reaching up to 100% for systems such as EP‐VAN‐AP/MNA or EP‐VAN‐AP/HHPA. This confirms that the materials are fully cross–linked and that the thermal program applied for cross–linking is appropriate for these systems.

**Table 2:** Physical and thermo‐mechanical properties of the Schiff‐based thermosets developed with anhydrides.

Schiff‐based epoxy	Anhydride	Apparent Density [g cm^−3^]	Gel Content [%]	Shore D [SD]	*E*’ glassy region [GPa]	*E*’ rubbery region [MPa]	*υ* (mmol∙cm^−3^)	tan *δ* amplitude	*T*_a_ [°C]
EP‐VAN‐AP	GA	1.24 ± 0.11	99.78 ± 0.02	83 ± 0.5	1.39 ± 0.002	25 ± 2	2.47 ± 0.01	0.17 ± 0.02	155 ± 3
	MA	1.05 ± 0.13	99.7 ± 0.03	86 ± 0.8	1.49 ± 0.001	31 ± 4	3.32 ± 0.01	0.24 ± 0.02	156 ± 4
	SA	1.22 ± 0.15	99.15 ± 0.02	88 ± 0.8	1.67 ± 0.001	46 ± 5	4.50 ± 0.02	0.14 ± 0.01	167 ± 3
	MNA	1.05 ± 0.14	100 ± 0.01	83 ± 1	0.79 ± 0.003	3 ± 0.5	0.30 ± 0.02	1.17 ± 0.01	117 ± 5
	HHPA	1.25 ± 0.11	100 ± 0.01	88 ± 0.7	1.13 ± 0.002	1 ± 0.2	0.29 ± 0.02	1.23 ± 0.03	108 ± 5
	THPA	1.15 ± 0.10	99.71 ± 0.04	85 ± 0.9	1.74 ± 0.003	19 ± 3	1.87 ± 0.01	0.17 ± 0.01	168 ± 3
	DDSA	1.13 ± 0.13	98.75 ± 0.05	83 ± 0.9	1.21 ± 0.005	12 ± 2	1.09 ± 0.01	0.30 ± 0.03	125 ± 2
	HMPA	1.19 ± 0.12	99.65 ± 0.05	84 ± 0.8	1.29 ± 0.004	6 ± 0.8	0.65 ± 0.03	0.79 ± 0.02	129 ± 2
EP‐VAN‐DiAP	GA	0.85 ± 0.13	99.59 ± 0.05	86 ± 0.5	0.53 ± 0.002	26 ± 4	1.84 ± 0.01	0.26 ± 0.02	197 ± 4
	MA	0.89 ± 0.15	99.65 ± 0.04	93 ± 0.5	0.65 ± 0.001	20 ± 3	2.45 ± 0.02	0.43 ± 0.01	334 ± 3
	SA	0.98 ± 0.16	99.60 ± 0.05	91 ± 0.5	0.57 ± 0.002	40 ± 5	2.67 ± 0.02	0.20 ± 0.01	252 ± 4
	MNA	1.03 ± 0.15	99.45 ± 0.05	90 ± 0.8	2 ± 0.005	32 ± 5	3.12 ± 0.01	0.27 ± 0.02	249 ± 4
	HHPA	1.04 ± 0.18	99.67 ± 0.04	88 ± 1	1.2 ± 0.003	15 ± 2	4.23 ± 0.03	0.24 ± 0.03	218 ± 5
	THPA	0.88 ± 0.10	100 ± 0.01	89 ± 1	0.96 ± 0.001	17 ± 4	3.66 ± 0.02	0.18 ± 0.03	222 ± 5
	DDSA	1.25 ± 0.16	98.86 ± 0.02	83 ± 0.8	1.2 ± 0.001	6 ± 1	0.72 ± 0.01	0.47 ± 0.01	114 ± 3
	HMPA	1.04 ± 0.15	100 ± 0.01	88 ± 0.9	1.3 ± 0.004	8 ± 1	2.22 ± 0.01	0.42 ± 0.02	202 ± 4
EP‐SYR‐AP	GA	1.28 ± 0.14	99.71 ± 0.02	85 ± 0.8	1.39 ± 0.005	9 ± 1	0.87 ± 0.02	0.40 ± 0.02	113 ± 2
	MA	1.18 ± 0.13	99.87 ± 0.02	88 ± 1	1.49 ± 0.005	12 ± 2	1.20 ± 0.02	0.48 ± 0.01	126 ± 2
	SA	1.30 ± 0.11	99.68 ± 0.03	83 ± 1	1.67 ± 0.004	26 ± 4	2.22 ± 0.03	0.37 ± 0.01	131 ± 2
	MNA	1.36 ± 0.17	99.40 ± 0.05	82 ± 1	0.80 ± 0.003	1 ± 0.2	0.25 ± 0.01	0.92 ± 0.03	105 ± 1
	HHPA	1.25 ± 0.15	99.89 ± 0.02	81 ± 0.9	1.13 ± 0.005	1 ± 0.1	0.18 ± 0.01	1.04 ± 0.01	96 ± 1
	THPA	1.19 ± 0.11	99.74 ± 0.04	84 ± 0.8	1.74 ± 0.001	2 ± 0.2	0.41 ± 0.02	0.94 ± 0.02	113 ± 2
	DDSA	1.08 ± 0.10	99.82 ± 0.02	84 ± 0.8	1.21 ± 0.001	4 ± 0.3	0.53 ± 0.01	1.08 ± 0.02	91 ± 1
	HMPA	1.08 ± 0.13	99.53 ± 0.05	83 ± 0.9	1.29 ± 0.002	1 ± 0.2	0.25 ± 0.02	1.18 ± 0.02	107 ± 3
EP‐SYR‐DiAP	MNA	1.22 ± 0.19	99.46 ± 0.03	87 ± 0.7	0.61 ± 0.005	0.5 ± 0.1	0.14 ± 0.02	0.89 ± 0.01	105 ± 3
	HHPA	1.07 ± 0.12	99.6 ± 0.04	83 ± 0.8	0.36 ± 0.003	0.8 ± 0.1	0.15 ± 0.02	0.73 ± 0.03	136 ± 4
	THPA	1.12 ± 0.11	99.35 ± 0.05	80 ± 1	0.30 ± 0.003	2 ± 0.4	0.23 ± 0.01	0.68 ± 0.03	112 ± 3
	DDSA	1.09 ± 0.10	99.80 ± 0.02	81 ± 1	0.78 ± 0.004	3 ± 0.4	0.30 ± 0.01	0.83 ± 0.01	112 ± 3
	HMPA	1.20 ± 0.11	99.69 ± 0.03	89 ± 0.5	0.86 ± 0.002	1 ± 0.1	0.20 ± 0.01	1.04 ± 0.02	112 ± 4

### 2.2 Thermo‐Mechanical Behavior of the Poly(ester‐imine) Thermosets

**Figure 3:**
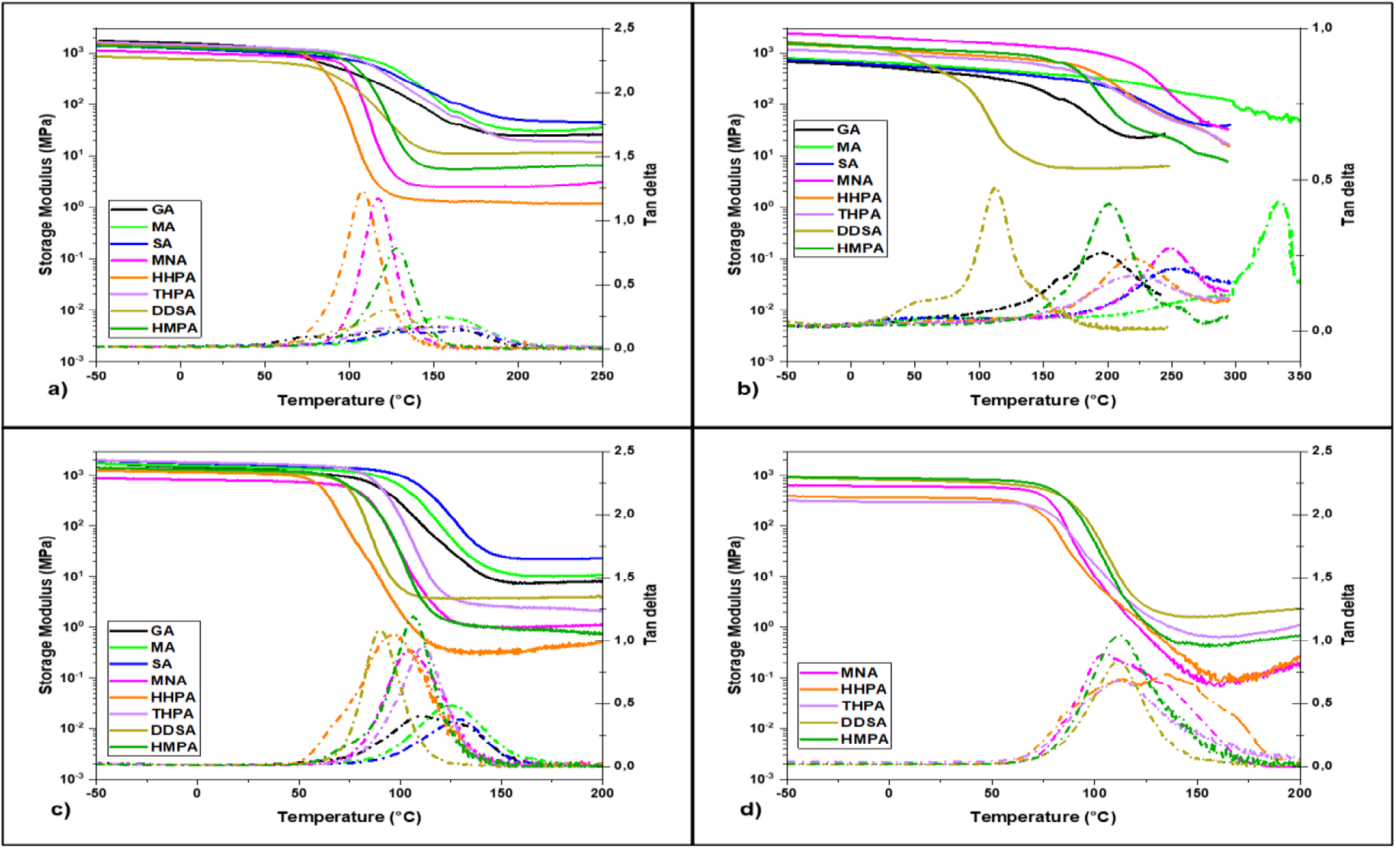
Storage moduli (E’; solid line) and damping factor (tan *δ*; dotted line) of the a) EP‐VAN‐AP, b) EP‐VAN‐DiAP; c) EP‐SYR‐AP, and d) EP‐SYR‐DiAP based thermosets with anhydrides.

The evolution of viscoelastic properties, including storage modulus (*E*') and damping factor (*tan* δ), as a function of temperature is illustrated in **Figure** **3**. The key thermomechanical parameters, including the storage modulus in the glassy region and the maximum damping factor (*T*_a_) values for each sample, are provided in Table 2.

In the glassy region, the systems developed with EP‐VAN‐AP and EP‐SYR‐AP exhibit high storage modulus values, ranging between 0.8 and 1.74 GPa, indicating greater rigidity compared to the systems based on EP‐SYR‐DiAP which show lower *E*' values, ≈0.3‒0.9 GPa. For systems based on EP‐VAN‐DiAP, the storage modulus varies from 0.53 GPa (EP‐VAN‐DiAP/GA) to 2 GPa (EP‐VAN‐DiAP/MNA), with the specific value being influenced by the anhydride used as curing agent. Lower *E’* values indicate that these materials are suitable for flexible electronic components or as soft and pliable biomedical devices, including catheters, prosthetic liners, and flexible tubing. Additionally, they are well‐suited for automotive interior components such as dashboards, door panels, and trims.^[^ ^48^, ^49^, ^50^ ^]^

The thermosets which exhibit *E*' at 25 °C ranging from 0.8 to 2 GPa, are suitable for structural applications such as brackets, frames, and panels in the aerospace and automotive industries. These materials can also be used in the fabrication of printed circuit boards or as matrices for high‐performance composites used in marine applications (e.g., boat hulls) and wind turbine blades.^[^ ^48^, ^49^, ^50^ ^]^ According to Table 2, the storage modulus in the rubbery region varies from 1 to 50 MPa, depending on the formulation's composition.

We can notice that for the EP‐VAN‐AP the highest *E*' values in the rubbery region are those of thermosets produced with SA, MA, and GA anhydrides ≈46, 31, and 25 MPa respectively. A similar trend is observed in EP‐SYR‐AP systems, where these anhydrides yield materials with *E*' values at 180 °C of ≈26, 12, and 9 MPa. These results can be correlated with shorter segment lengths between cross–links and higher cross–link densities generated by the opening of SA, MA, and GA anhydrides, as indicated in Table 2. The DDSA anhydride follows this trend in both EP‐VAN‐AP and EP‐SYR‐AP systems. Conversely, the lowest *E*' values in the rubbery region were obtained when cyclic substituted anhydrides such as HHPA or MNA were used as hardeners. Surprisingly, despite its structural similarity to HHPA, THPA produced materials with higher *E’* values, in all EP‐VAN‐AP and EP‐SYR‐AP systems, regardless of whether they were in the glassy or rubbery region, as well as in the rubbery region for EP‐SYR‐DiAP materials.

In industrial applications, the glass transition value (*T*_g_) is a critical parameter for defining the operational temperature range of polymeric materials.^[^ ^51^ ^]^ In this study, the *T*_g_ of the thermosets was determined via DMA at the temperature corresponding to the peak of the damping factor (*tan* δ), denoted as *T*_a_. Figure 3 illustrates the evolution of *tan* δ with temperature, while its amplitude reflects the material's energy dissipation capacity, which is associated with molecular mobility within the polymer network.^[^ ^52^ ^]^ Based on the *T*_a_ values, the glass transition values of the Schiff‐based thermosets vary from 90 to nearly 335 °C. The most rigid systems are those based on EP‐VAN‐DiAP, exhibiting *T*_a_ values ranging from 114 to 334 °C, being followed by EP‐VAN‐AP materials, with *T*_a_ values between 108‒168 °C. These are succeeded by EP‐SYR‐DiAP systems that have *T*_a_ values between 105 °C and 136 °C, and EP‐SYR‐AP with *T*_a_ ranging from 91 to 131 °C. This variation in *T*_a_ values indicates the potential of these systems for applications requiring different thermal performance levels. The reported *T*_a_ values are comparable to those of commercial epoxy resins such as EPON Resin 828 (*T*_g_ = 125‒130 °C), D.E.R. 331 Epoxy Resin (*T*_g_ = 115‒125 °C), EPIKOTE Resin 862 (*T*_g_ = 90 °C), and Araldite GY 6010 (*T*_g_ = 90‒95 °C). When comparing these data with literature reports on Schiff‐based epoxy thermosets (Table S1, Supporting Information), certain formulations demonstrate superior performances that can be attributed to the use of diamine curing agents, which promote higher cross–linking densities due to a higher functionality of diamines compared with that of anhydrides.^[^ ^53^ ^]^ A key difference between thermosets cured with diamines and those cured with anhydrides is the nature of the resulting polymer network. The curing reaction of epoxies with diamines generates polyethers with hydroxyl groups that can increase the cross–link density by opening the oxirane rings or they can form hydrogen bonds with other hydroxyl or amino groups within the polymer network. This additional hydrogen bonding further strengthens the network structure enhancing material mechanical properties.

The Shore D hardness tests of the thermosets were also assessed, yielding values characteristic of rigid materials, (80‒93 SD). These results indicate that the materials possess high hardness and are well‐suited for applications requiring high mechanical strength and resistance to deformation. Materials with similar Shore D values are commonly used in demanding engineering applications due to their excellent durability and wear resistance. The developed thermosets exhibit hardness values comparable to commercial epoxy resins such as West System 105 Epoxy Resin (83 SD) and Sikadur‐52 (85 SD).

The apparent density of the developed systems, as presented in Table 4, ranges from 0.85 to 1.36 g cm^−^^3^, suggesting their suitability for lightweight applications across various industries. Apparent density, which is determined by the mass‐to‐volume ratio, accounts for material porosity and structural imperfections, making it a relevant indicator of performance in real‐world applications. Many industrial materials exhibit some level of porosity or surface irregularity due to manufacturing processes, and apparent density depicts these imperfections, making it more representative of how the material performs in end‐use conditions. The low density of these thermosets highlights their potential for weight reduction in aerospace, automotive, marine, and sporting goods applications. Their density values are comparable to those of commercial epoxy resins such as Araldite LY 5052 / Aradur 5052 (ρ = 1.15 g cm^−3^), West System 105 Epoxy Resin (ρ = 1.15 g cm^−3^), and Sikadur‐31 CF Normal (ρ = 1.35 g cm^−3^). The density values determined for these bio‐based systems confirm their potential as viable alternatives to traditional commercial resins in lightweight and performance‐critical applications.

The structure of the Schiff‐base compounds plays a significant role in determining the structure of polymer networks and their thermo‐mechanical properties. EP‐VAN‐AP contains a single methoxy group attached to its aromatic ring, whereas EP‐SYR‐AP and EP‐VAN‐DiAP feature two methoxy groups, and EP‐SYR‐DiAP has four. The presence of methoxy substituents in these rigid structures alters the molecule's polarizability and affects molecular packing, as methoxy units extend out of the molecular plane.^[^ ^54^, ^55^ ^]^ This projection increases the intermolecular distance between polymer chains, reducing the Van der Waals interactions between chains. Comparing EP‐SYR‐AP and EP‐SYR‐DiAP, an additional factor emerges‒ the molecular symmetry and length of the molecule. EP‐SYR‐DiAP is a symmetric molecule, which mitigates the disruptive effects of methoxy groups. This balancing effect is reflected in the thermo‐mechanical properties (Table 2), where EP‐VAN‐AP‐based thermosets outperform EP‐SYR‐AP‐based ones, confirming the disruptive influence of methoxy groups. For EP‐SYR‐DiAP‐based thermosets, similar or slightly higher *T*_g_ values are observed, despite their lower molecular density. This can be explained by the larger molecular size of EP‐SYR‐DiAP, which compensates for the drawbacks introduced by the methoxy groups through their structural symmetry. When considering EP‐VAN‐DiAP, a symmetrical structure similar to EP‐SYR‐DiAP but with only two methoxy groups, the influence of these structural features becomes even more apparent. EP‐VAN‐DiAP combines the beneficial effects of a lower methoxy content (reducing the disruptive impact on polymer packing) with its symmetric structure, enhancing its overall thermo‐mechanical properties. As a result, EP‐VAN‐DiAP‐based thermosets outperform those based on EP‐SYR‐DiAP, as they achieve an optimal balance between rigidity and molecular packing, maintaining strong physical interactions between polymer chains.

The thermo‐mechanical properties of these systems are also influenced by the structure of the anhydrides used as curing agents. Two types of anhydrides were used in this study: i/ those with simple structures (e.g., GA, MA, SA), and ii/ those with more complex structures containing additional rings or chains (e.g., MNA, THPA, HHPA, HMPA, DDSA). Anhydrides with simpler structures exhibit better thermo‐mechanical properties, which can be attributed to their smaller number of atoms, resulting in their shorter intermolecular distances between linkages. Conversely, anhydrides with more complex structures lead to lower molecular density, lower glass transition temperatures, and reduced storage modulus. The additional cyclic structures or chain substituents in these anhydrides increase the distance between molecules, creating voids where physical forces are insufficient to establish strong interactions.^[^ ^56^ ^]^ Notably, the presence of double bonds within the anhydride structure enhances the thermo‐mechanical properties compared to structurally similar anhydrides without double bonds, such as MA and SA or THPA and HHPA. The double bonds contribute to the planarity of the molecule and increase its electronic density, improving both physical interactions between molecules and their overall stability.^[^ ^57^ ^]^ Consequently, the inclusion of double bonds strengthens the network, leading to superior performance in terms of glass transition temperature and mechanical moduli.

### 2.3 Thermal Stability And Moisture Behavior of the Poly(ester‐imine) Thermoset

**Figure 4:**
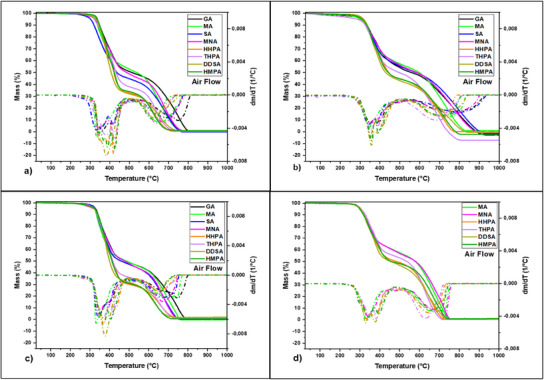
TGA and DTG curves vs temperature of the a) EP‐VAN‐AP, b) EP‐VAN‐DiAP; c) EP‐SYR‐AP, and d) EP‐SYR‐DiAP based thermosets in oxidative atmosphere, heating rate 10 °C per min.

To gain a thorough understanding of the decomposition behavior and thermal properties of the developed polymers, TGA was conducted in both air and nitrogen atmospheres. This dual approach provided a comprehensive perspective on the materials' responses under different environmental conditions. **Figures** **4** and S5 (Supporting Information) present the thermal profiles of the studied thermosets, with relevant data summarized in **Table** **3**. The TGA and DTG curves in the nitrogen atmosphere are shown in Figure S5, Supporting Information.

**Table 3:** Thermogravimetric data obtained for Schiff‐base thermosets in oxidative atmosphere.

Schiff‐base epoxy	Anhydride	T_5%_ [°C]	T_30%_ [°C]	T_dmax_ [°C]	T_s_ [°C]
EP‐VAN‐AP	GA	335 ± 5	399 ± 3	359 ± 2	183 ± 1.3
	MA	328 ± 4	388 ± 4	340 ± 3	178 ± 1.2
	SA	305 ± 5	370 ± 5	330 ± 3	169 ± 1.1
	MNA	329 ± 5	398 ± 5	340 ± 3	181 ± 1.1
	HHPA	339 ± 4	385 ± 4	418 ± 5	180 ± 1.5
	THPA	322 ± 3	388 ± 4	418 ± 5	177 ± 1.3
	DDSA	329 ± 3	378 ± 4	379 ± 4	176 ± 1.4
	HMPA	335 ± 4	385 ± 5	428 ± 4	179 ± 1.1
EP‐VAN‐DiAP	GA	312 ± 3	383 ± 5	350 ± 4	174 ± 1.4
	MA	301 ± 3	388 ± 3	346 ± 2	173 ± 1.3
	SA	312 ± 4	389 ± 3	355 ± 3	176 ± 1.4
	MNA	311 ± 3	399 ± 5	349 ± 3	178 ± 1.2
	HHPA	310 ± 2	381 ± 4	350 ± 3	173 ± 1.3
	THPA	286 ± 2	382 ± 4	354 ± 5	168 ± 1.2
	DDSA	318 ± 4	376 ± 2	356 ± 5	173 ± 1.3
	HMPA	310 ± 5	384 ± 2	354 ± 5	174 ± 1.4
EP‐SYR‐AP	GA	325 ± 4	385 ± 3	350 ± 4	177 ± 1.3
	MA	325 ± 4	370 ± 4	330 ± 4	173 ± 1.3
	SA	324 ± 5	375 ± 5	349 ± 2	174 ± 1.4
	MNA	315 ± 4	385 ± 4	359 ± 3	175 ± 1.5
	HHPA	326 ± 5	375 ± 3	369 ± 3	174 ± 1.4
	THPA	320 ± 4	375 ± 3	370 ± 3	173 ± 1.3
	DDSA	323 ± 4	368 ± 5	379 ± 5	172 ± 1.2
	HMPA	325 ± 3	375 ± 5	400 ± 5	174 ± 1.4
EP‐SYR‐AP	MNA	300 ± 2	380 ± 4	340 ± 2	171 ± 1.1
	HHPA	301 ± 2	372 ± 5	340 ± 2	168 ± 1.2
	THPA	305 ± 4	380 ± 3	379 ± 4	172 ± 1.2
	DDSA	305 ± 4	369 ± 3	335 ± 3	168 ± 1.2
	HMPA	298 ± 2	370 ± 5	350 ± 4	167 ± 1.3

Thermal degradation was evaluated by identifying the temperature at which the materials lost 5% of their mass (*T*_5%_), which marks the beginning of decomposition. In oxidative environments, the temperature at which 5% of the material's mass is lost (*T*_5%_) is significantly high, ranging from 286 to 339 °C. The thermal stability of Schiff‐based thermosets is influenced by the binding energy of the imine bond, which is relatively low. The number of imine bonds slightly affects the thermal stability of the resulting thermosets. Schiff‐base monomers with only one imine bond per molecule tend to form thermosets with higher thermal stability compared to those with two imine groups.[4,58,59] For example, systems with one imine group per epoxy molecule, such as EP‐VAN‐AP and EP‐SYR‐AP, show higher thermal stability (*T*_5%_ = 305‒335 °C) compared to systems with two imine groups per monomer molecule, as EP‐VAN‐DiAP and EP‐SYR‐DiAP, which lose 5% of their mass at slightly lower temperatures, ≈286‒318 °C. This difference in thermal stability is attributed to the reduced susceptibility of materials with fewer imine bonds to thermal degradation. Fewer imine bonds in the molecular structure mean there are fewer weak points that can break down during thermal exposure, thus enhancing the thermal stability of the thermosets.

The statistical heat resistance index (*T*_s_) provides a comprehensive measure of the material's thermal stability by considering the temperatures at which significant mass loss occurs during thermal degradation. It is calculated using the equation:^[^ ^28^, ^60^ ^]^

(1) $$T_{s}=0.49\left[T_{5\%}+0.6\left(T_{30\%}-T_{5\%}\right)\right]$$

Here, *T_5_*_%​_ is the temperature at which the sample loses 5% of its initial mass, and *T*_30%​_ is the temperature at which 30% mass loss occurs. This index not only captures the onset of thermal decomposition (*T*_5%_) but also considers the broader temperature range where substantial degradation occurs (from *T*_5%_ to *T*_30%_). For the EP‐VAN‐AP and EP‐SYR‐AP systems, the *T*_s_ values ranged from 169 to 183 °C, indicating robust heat resistance. Similarly, for EP‐VAN‐DiAP and EP‐SYR‐DiAP, *T*_s_ values ranged from 167 to 178 °C. These values suggest that EP‐VAN‐AP and EP‐SYR‐AP exhibit slightly higher heat resistance compared to EP‐VAN‐DiAP and EP‐SYR‐DiAP, likely due to differences in their chemical structures and thermal decomposition behaviors. The *T*_s_ index is crucial for evaluating materials intended for applications where thermal stability is paramount, such as aerospace components, automotive parts, or electronic devices that operate at elevated temperatures. It offers insights into how these thermosets will perform under thermal stress and helps in selecting the most suitable material based on its thermal resilience characteristics.

The thermal degradation of the Schiff‐base thermosets in an oxidative atmosphere follows a complex pattern characterized by two distinct stages. The initial stage begins with an onset temperature ranging from 280 to 330 °C. This temperature range marks the point at which the material starts to undergo significant decomposition, primarily due to the pyrolysis of the network structure formed by the Schiff base. During this stage, the material presents a substantial mass loss, typically between 50% and 69%. As the temperature increases, the degradation process accelerates, reaching a maximum degradation rate ≈360‒390 °C. This stage concludes as the temperature reaches ≈470‒550 °C. Oxidative conditions promote the breaking of imine bonds within the network structure, initiating the release of volatile components and the formation of charred residues.^[^ ^44^, ^58^ ^]^ Following the first stage, the degradation process transitions to a second stage at higher temperatures. This stage begins ≈500‒550 °C and continues until complete degradation occurs ≈800 °C. The maximum temperature of the decomposition peak in this stage is observed between 630 and 730 °C, with a mass loss ranging from 31% to 50%. During the second stage, thermal oxidation processes continue to degrade the residual structures formed during the previous stages of decomposition.

**Figure 5:**
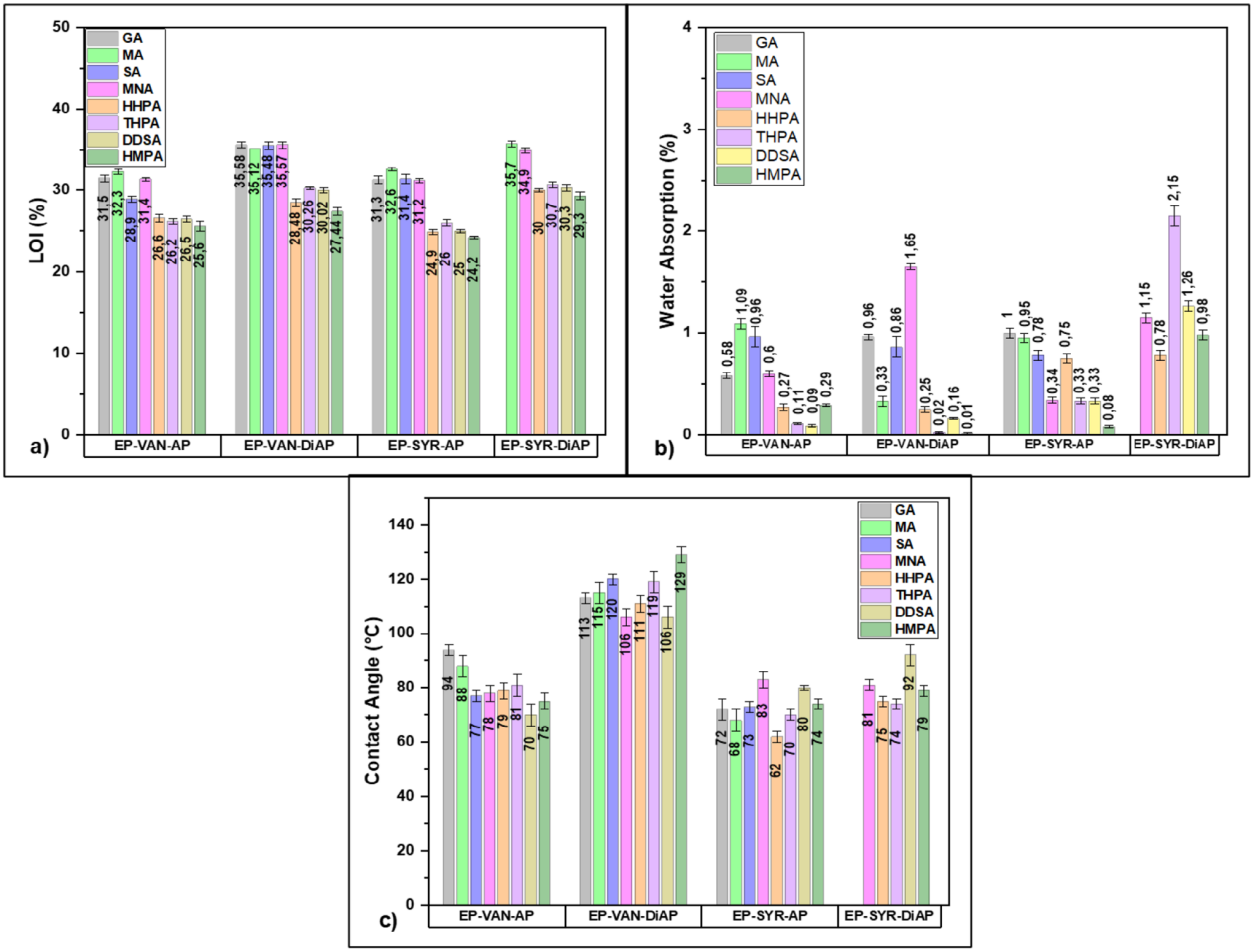
Graphical representation of a) LOI, b) Water absorption, and c) Contact angle values for the Schiff‐base thermosets.

The thermal stability and flame‐retardant properties of Schiff‐base resins were also studied by calculating the limiting oxygen index (*LOI*). It measures the minimum concentration of oxygen required to sustain the combustion of a material. The *LOI* is calculated using the formula:^[^ ^61^, ^62^ ^]^

(2) $$LOI=17.5+0.4\times Cy_{850}$$

where *C*_y850_ represents the carbon yield at 850 °C in an inert atmosphere. The obtained data are plotted in **Figure** **5**a.

The materials exhibited *LOI* values ranging from ≈24% to 36%. Schiff‐base thermosets tend to exhibit intrinsically high *LOI* values due to the presence of nitrogen atoms.^[^ ^4^, ^63^ ^]^ Then, the thermosets based on EP‐VAN‐AP and EP‐SYR‐AP, containing a single imine group per monomer molecule, typically show slightly lower *LOI* values (24% to 32%) compared to those based on EP‐VAN‐DiAP and EP‐SYR‐DiAP, that contains two imine groups and demonstrate *LOI* values ranging from ≈27% to 36%. Compared to literature data (Table S1, Supporting Information), the limiting oxygen index (*LOI*) values for Schiff‐based epoxy systems designed in this study are similar to those cured with diamines. Comparable flame‐retardant properties across these different curing systems emphasize the inherent flame‐retardant effect of the Schiff‐base structure itself.^[^ ^64^, ^65^ ^]^ In conclusion, the developed Schiff‐base thermosets demonstrate good thermal stability and flame‐retardant characteristics, as indicated by their LOI values derived from TGA analyses.

These properties make them suitable for applications where fire safety and resistance to combustion are critical considerations. For example, these thermosets can be used in aerospace components, and electrical insulation, providing flame protection in critical environments. These materials are also valuable in the automotive industry for interior and structural components, as well as in construction for flame‐retardant panels and adhesives. Additionally, they can be incorporated into protective textiles for firefighter suits and marine applications to meet stringent safety regulations. While the LOI values of Schiff‐base thermosets indicate promising flame‐retardant characteristics, it is important to note that specialized tests are required for each specific application to certify materials as fire‐resistant. The LOI serves as an initial parameter, providing a general indication of the material's potential for flame retardancy. However, to fully assess their fire safety properties and determine their suitability for particular applications, further in‐depth testing tailored to the specific conditions of use is essential.

The hydrostability of the developed poly(ester‐imine) thermosets was investigated through water absorption measurements. The water absorption tests involved immersing the samples in water at room temperature for 24 h, with the results presented in Figure 5b. Moisture behavior in polymeric materials is influenced by both physical and chemical factors, such as manufacturing protocols, potential manufacturing defects, cross–linking density, and the presence of hydrophilic functional groups.^[^ ^66^, ^67^, ^68^ ^]^ According to the obtained results (Figure 5b), the developed materials exhibit very low water absorption after 24 h of immersion, ranging from 0.01% to 1.65%. The exception to this behavior is the EP‐SYR‐DiAP/ THPA thermoset, which exhibits a higher *WA*% ≈2.15%. The water absorption capacity of this system is influenced by the degree of cross–linking within the polymeric structure. As previously described by the gel content (GC), the degree of cross–linking directly impacts the amount of water absorbed by the material. Typically, a lower degree of cross–linking results in higher water absorption. Therefore, the highest water absorption values obtained for these materials correlate with their slightly lower cross–link density.

The wettability of the polymeric materials was further investigated using contact angle measurements, and the results are graphically represented in Figure 5c. The contact angle test assesses the hydrophilicity or hydrophobicity of the material's surface, which is crucial for applications involving surface coatings and interactions with water. According to international standards (ASTM D7334 and ASTM D5946), a surface is considered hydrophilic if the contact angle is <45°, hydrophobic if the angle is >90°, and intermediate if the contact angle ranges from 45° to 90°. The designed Schiff‐base thermosets displayed contact angle values between 62° and 129°, indicating intermediate wettability behavior that leans more toward hydrophobicity. The systems based on EP‐VAN‐DiAP and the EP‐VAN‐AP/GA, EP‐VAN‐AP/MA, EP‐SYR‐AP/MNA thermosets exhibited a combination of low water absorption and moderate to high contact angles, suggesting that these materials possess good hydrostability, making them suitable for various applications, including outdoor protective coatings and moisture‐resistant components.

## 3 Conclusions

Traditional petrochemical‐derived resins contribute to environmental degradation through the release of toxic chemicals and greenhouse gases during their production and disposal. In contrast, the biobased resins developed in this study are synthesized from renewable resources, such as vanillin and syringaldehyde, significantly reducing the carbon footprint associated with their lifecycle. The use of eco‐friendly anhydrides for cross–linking further minimizes the environmental impact, as these substances are less hazardous and more biodegradable than their conventional counterparts. The materials developed in this study demonstrate a wide range of properties, making them suitable for high‐end industrial applications. Dynamic Mechanical Analysis (DMA) data provided valuable insights into the viscoelastic properties of these materials. The storage modulus (*E'*) at 25 °C ranged from 0.3 to 2 GPa, suggesting that the materials are versatile for applications ranging from flexible electronic components to rigid structural parts. The *tan* delta peaks indicate glass transition values between 90 °C and 334 °C, highlighting the robust thermal performance of these resins. Shore D hardness values, ranging from 80 to 93 SD, categorize these materials as extra‐hard, making them ideal for high‐durability applications, such as automotive parts and aerospace components. Additionally, the density values ranging from 0.85 to 1.36 g cm^−^^3^ highlight their suitability for lightweight applications, where a high strength‐to‐weight ratio is crucial. Thermogravimetric Analysis (TGA) revealed high thermal stability, with *T*_5%_ values in oxidative environments ranging from 280 to 330 °C. This indicates a strong resistance to thermal degradation, making the materials suitable for high‐temperature applications. Furthermore, the statistical heat resistance index (*T*_s_) yielded values between 167 and 183 °C, further confirming the thermal robustness of these resins. Water absorption tests demonstrated low moisture uptake, with most materials absorbing between 0.01% and 1.65% water after 24 hours of immersion. Wettability measurements, evaluated by contact angle tests, showed values between 62° and 129°, suggesting a tendency toward hydrophobic behavior, which is advantageous for applications requiring resistance to water and moisture. According to international standards, these contact angles indicate moderately hydrophobic surfaces, making them suitable for coatings and outdoor applications.

In summary, this study successfully developed biobased Schiff base thermosetting epoxy resins that exhibit excellent thermal stability, low water absorption, favorable wettability, significant stiffness, and adequate durability. These properties make them versatile for high‐performance aerospace and space applications, contributing to environmental sustainability by reducing dependence on petrochemical‐derived materials and lowering the overall carbon footprint.

The dual structural feature of these thermosets, combining the chemical resistance and thermal stability of esters with the functionality of imines, contributes to their unique properties.

## 4 Experimental Section

### Materials

Vanillin aldehyde (VAN), syringaldehyde (SYR), *p*‐phenylenediamine (DiAP), *p*‐aminophenol (AP), tetrahydrofuran (THF), dichloromethane (DCM), ethanol, epichlorohydrin (ECH), tetrabutylammonium chloride (TBAC), and sodium hydroxide (NaOH) were used for the synthesis and subsequent Schiff bases glycidylation. The Schiff based epoxy monomers, laboratory synthesized and characterized by H‐NMR, FT‐IR,^[^ ^31^ ^]^ were cross–linked with various anhydrides, including glutaric anhydride (GA), itaconic anhydride (IA), maleic anhydride (MA), succinic anhydride (SA), citraconic anhydride (CA), methyl nadic anhydride (MNA), hexahydrophthalic anhydride (HHPA), *cis*‐1,2,3,6‐tetrahydrophthalic anhydride (THPA), dodecenylsuccinic anhydride (DDSA), and hexahydro‐4‐methylphthalic anhydride (HMPA). All reagents were acquired from Sigma Aldrich France and used without further purification. The molecular structures of these compounds are detailed in **Tables** **4** and **5**.

**Table 4:** Molecular structure of the Schiff‐based epoxy monomers.

Acronym	Chemical Structure	Acronym	Chemical Structure
EP‐VAN‐AP	2	EP‐VAN‐DiAP	2
EP‐SYR‐AP	2	EP‐SYR‐DiAP	2

**Table 5:** Molecular structure of the anhydrides used as hardeners.

Acronym	Chemical Structure	Acronym	Chemical Structure
Glutaric Anhydride (GA)	2	Methyl Nadic Anhydride (MNA)	2
Itaconic Anhydride (IA)	2	Hexahydro Phthalic Anhydride (HHPA)	2
Maleic Anhydride (MA)	2	*cis*‐1,2,3,6‐ Tetrahydrophthalic Anhydride (THPA)	2
Succinic Anhydride (SA)	2	Dodecenylsuccinic Anhydride (DDSA)	2
Citraconic Anhydride (CA)	2	Hexahydro‐4‐Methyl Phthalic Anhydride (HMPA)	2

### Thermosets Samples Preparation

The preparation of thermoset samples was achieved by cross–linking the previously synthesized epoxy Schiff‐based monomers with various anhydrides in a stoichiometric molar ratio of 1:1 (epoxy group to anhydride group). Initially, the stoichiometric mixture of epoxy monomer and anhydride hardener was thoroughly mechanically homogenized using a mortar. Then, the mixture was transferred to a beaker and heated at a temperature ≈65‒70 °C for 5 minutes, with continuous stirring, on a heating plate. The resulting homogeneous mix was then poured into silicon molds for thermal cross–linking. The curing process followed a standardized thermal protocol for all systems. The samples were isothermally heated in an oven at 120 °C for 2 h, followed by another isotherm at 150 °C for 3 h. For post‐curing, the samples were subjected to 180 °C for 2 h, then at 230 °C for 1 h. After completion of the thermal curing protocol, the cross–linked samples were removed from the oven, cooled to room temperature (RT), and subsequently analyzed.

### Attenuated Total Reflection Fourier Transform Infrared Spectroscopy (ATR‐FTIR)

The structural evolution during cross–linking was monitored using Fourier Transform Infrared (FTIR) spectroscopy. These analyses were conducted with a Nicolet iS50 FT‐IR spectrometer in attenuated total reflectance (ATR) mode, employing a PIKE GladiATR single diamond system. To investigate the structural changes on the reactive systems during cross–linking, fresh mixtures were subjected to a 10 °C per min dynamic heating from 30 to 250 °C. Spectra were collected every 20 °C and were registered over a wavenumber range of 4000‒500∙cm^−1^, employing 32 scans and a resolution of 4∙cm^−1^, with the air spectrum recorded as the background.

### Differential Scanning Calorimetry (DSC)

A Mettler Toledo DSC 3 instrument was operated to monitor the cross–linking behavior of Schiff‐based thermosetting mixtures. Samples weighing ≈6‒8 mg were placed into 40 µL aluminum DSC pans. The thermal analysis was conducted by recording the heat flow variation as a function of temperature for the freshly prepared mixtures. The temperature range for this study spanned from 25 to 300 °C, with a constant heating rate of 10 °C/ min. This method enabled the detailed observation of exothermic transitions associated with the cross–linking process. For better accuracy, three samples were tested for each formulation.

### Apparent Density

The apparent density of the materials was determined by calculating the mass‐to‐volume ratio using five rectangular specimens (50 × 5 × 4 mm^3^) for each sample. Initially, the volume of each specimen was calculated. Subsequently, their mass was measured using a precision Mettler Toledo ML3002T balance. The obtained values for mass and volume were averaged to minimize the measurement errors, ensuring more accurate density calculations.

### Dynamic mechanical analysis (DMA)

A Mettler Toledo DMA 1 instrument, equipped with a Single Cantilever fixture, was employed to investigate the thermo‐mechanical properties of Schiff‐based thermosets. The analyses were conducted in a temperature range from −50 to 250 °C, with a controlled heating rate of 3 °C per min. DMA was performed to measure the storage modulus, loss modulus, and tan *d* of the thermoset samples. The test frequency was maintained at 1.0 Hz, and the displacement amplitude was set to 20 µm to ensure accurate characterization of the viscoelastic behavior. For better accuracy, two samples were tested for each formulation. To further analyze the thermoset materials, cross–link densities (*υ*) and the average molar weight of segments between cross–links (*M*_c_) were calculated using the following equations derived from Flory (1953) and Tobolsky (1960) studies:^[^ ^69^, ^70^ ^]^

(3) $$\upsilon =\frac{E^{\prime }}{3RT}\;M_{c}=\frac{3\rho RT}{E^{\prime }}$$

Where *E'* represents the storage modulus of the thermoset in the rubbery plateau region at *T*_g_ + 60 °C, *R* is the universal gas constant, *T* is the absolute temperature, and *ρ* is the density of the resin.

### Shore Hardness Tests

The measurement of the hardness of materials was conducted using a Zwick Roell 3116 device, employing an analog Shore D head to gauge penetration depth under a 50 N loading force. To ensure rigorous analysis, each formulation underwent five separate tests, and the average value was calculated.

### Thermogravimetric Analysis (TGA)

The thermal stability of the Schiff‐base thermosets was analyzed using a Mettler Toledo TGA 2 apparatus equipped with STAR Software. Cross–linked samples weighing ≈10‒12 mg were tested under oxidative (air) and inert (N_2_) atmospheres at a flow rate of 50 mL per min. The samples were heated from 25 to 1000 °C at a constant rate of 10 °C per min in alumina crucibles. The degradation temperature of the materials was determined as the temperature at which the samples lost 5% (*T*_5%_) of their initial mass. For better accuracy, three samples were tested for each formulation.

### Gel Content (GC, %)

For the evaluation of the thermosets’ gel content (*GC, %*), a solvent extraction method was employed. Rectangular samples measuring 10 × 10 × 4 mm^3^ were first conditioned in an oven at 50 °C for 24 h to stabilize their mass. The initial mass (*w*_0_) of each sample was then measured using a Mettler Toledo ML3002T precision balance. Subsequently, the samples were fully immersed in 10 mL of toluene and left at room temperature for 72 h to allow the solvent to extract soluble components. After the extraction period, the samples were removed from the solvent, dried at 50 °C for 24 h to remove any remaining solvent, and then re‐weighed (*w*_f_). The gel content of the thermosets was determined using the equation:^[^ ^71^ ^]^

(4) $$GC,\;\%=\frac{w_{f}}{w_{0}}\times 100$$

This method quantifies the fraction of the material that remains insoluble after extraction, providing insights into the cross–linking density and network formation within the thermosets. For better accuracy, three samples were tested for each formulation.

### Water Absorption (WA, %)

The water absorption capacity of the materials was evaluated following ASTM D570 standard procedures. Initially, conditioned samples were held at 50 °C for 24 h to stabilize their mass (*w*_0_). Subsequently, these samples were immersed in distilled water at room temperature. At intervals of 24 h, the thermoset samples were removed from the water, gently dried with filter paper to remove surface moisture, and then weighed to determine their wet mass (*w*_d_). The water absorption percentage (*WA*%) of the Schiff‐based thermosets was calculated by employing the equation:^[^ ^72^ ^]^

(5) $$WA,\;\%=\frac{W_{d}-W_{0}}{W_{0}}\times 100$$

This method allows for the measurement of water absorption characteristics over time, providing insights into the material's ability to absorb moisture under ambient conditions, up to saturation levels. For better accuracy, three samples were tested for each formulation.

### Water CONTACT Angle

The water contact angle measurements were performed at room temperature (24 ± 1 °C) using a free‐angle observation system Keyence VHX‐S650E equipped with Keyence VH‐Z100T wide‐range zoom lens (×100–×1000). A precise drop of distilled water was dispensed onto each sample using a syringe, ensuring uniform droplet size. Images of the droplets were captured immediately to minimize evaporation effects. For better accuracy, three measurements were recorded for each formulation.

## Conflict of Interest

The authors declare no conflict of interest.

## Supporting information



Supporting Information

## Data Availability

The data that support the findings of this study are available in the supplementary material of this article.

## References

[1] J. M. Millican , S. Agarwal , Macromolecules 2021, 54, 4455.

[2] D. K. Schneiderman , M. A. Hillmyer , Macromolecules 2017, 50, 3733.

[3] H. Sukanto , W. W. Raharjo , D. Ariawan , J. Triyono , M. Kaavesina , Open Engineering 2021, 11, 797.

[4] E. Desnoes , L. Toubal , A. H. Bouazza , D. Montplaisir , Polym. Eng. Sci. 2020, 60, 2593.

[5] D. Quan , Y. Ma , D. Yue , J. Liu , J. Xing , M. Zhang , R. Alderliesten , G. Zhao , Thin‐Walled Structures 2023, 186, 110671.

[6] R. Dallaev , T. Pisarenko , N. Papež , P. Sadovský , V. Holcman , Polymers (Basel) 2023, 15, 3964. 37836013 10.3390/polym15193964 PMC10574936

[7] M. D. N. Sakib , A. A. Iqba , Int. J. Automot. Mechan. Engin. 2021, 18, 3.

[8] B. C. Chakraborty , in Structural Adhesives, Wiley, Hoboken 2023, 397.

[9] Y. Ma , H. Liu , J. Wu , L. Yuan , Y. Wang , X. Du , R. Wang , P. W. Marwa , P. Petlulu , X. Chen , H. Zhang , Environ. Res. 2019, 176, 108575. 31299621 10.1016/j.envres.2019.108575

[10] X. Zhao , Y. Long , S. Xu , X. Liu , L. Chen , Y. Z. Wang , Mater. Today 2023, 64, 72.

[11] W. Zhao , J. Liu , S. Wang , J. Dai , X. Liu , Adv. Mater. 2024, 36, 2311242. 10.1002/adma.202311242 38504494

[12] V. K. Ponnusamy , D. D. Nguyen , J. Dharmaraja , S. Shobana , J. R. Banu , R. G. Saratale , S. W. Chang , G. Kumar , Bioresour. Technol. 2019, 271, 462. 30270050 10.1016/j.biortech.2018.09.070

[13] X. Lu , X. Gu , Int. J. Biol. Macromol. 2023, 229, 778. 36603715 10.1016/j.ijbiomac.2022.12.322

[14] R. Höfer , in Renewable Resources for Surface Coatings, Inks, and Adhesives, RSC Publication, Cambridge, 2023, 775.

[15] Y. Zhang , L. Mo , F. Chen , M. Lu , W. Dong , Q. Wang , F. Xu , F. Gu , Molecules 2014, 19, 2181. 24556615 10.3390/molecules19022181 PMC6271755

[16] M. Corbet , P. Metivier , F. Decampo , Process for production of vanillin and vanillin derivatives, WO2013166642, 2013.

[17] O. Ramaen , V. Sauveplane , R. Pandjaitan , EP 2 749 644 A1 2014.

[18] J. N. Murwanashyaka , H. Pakdel , C. Roy , Sep. Purif. Technol. 2001, 24, 155.

[19] S. Ota , K. Michishio , M. Harada , Mater. Today Commun. 2022, 31, 103501.

[20] J. Zhang , C. Jiang , G. Deng , M. Luo , B. Ye , H. Zhang , M. Miao , T. Li , D. Zhang , Nat. Commun. 2024, 15, 4869. 38849328 10.1038/s41467-024-49272-3 PMC11161517

[21] P. Zamani , O. Zabihi , M. Ahmadi , M. R. Zamani , M. J. Zohuriaan‐Mehr , T. Kannangara , P. Joseph , M. Naebe , Compos Part A Appl Sci Manuf 2024, 179, 108016.

[22] T. Yan , H. Jiang , W. Pang , T. He , M. Cheng , Z. Wang , C. Li , S. Sun , S. Hu , J. Appl. Polym. Sci. 2024, 141, app55684.

[23] Z. Li , Y. Dong , C. Li , H. Guan , L. Shen , J. Meng , K. Guo , React. Funct. Polym. 2024, 194, 105804.

[24] M. A. Rashid , M. N. Hasan , M. A. R. Dayan , M. S. Ibna Jamal , M. K. Patoary , Reactions 2023, 4, 66.

[25] F. C. Klein , M. Vogt , V. Abetz , Macromol. Mater. Eng. 2024, 309, 2300187.

[26] J. H. Emon , M. A. Rashid , M. A. Islam , M. N. Hasan , M. K. Patoary , Reactions 2023, 4, 737.

[27] J. Li , Z. Weng , Q. Cao , Y. Qi , B. Lu , S. Zhang , J. Wang , X. Jian , Chem. Eng. J. 2022, 433, 134512.

[28] C. Yang , X. Xia , Y. Xiao , G. Wei , W. Li , Y. Lu , Polym. Degrad. Stab. 2024, 221, 110666.

[29] H. Nabipour , S. Rohani , Y. Hu , Polym. Degrad. Stab. 2023, 214, 110410.

[30] H. Nabipour , X. Wang , L. Song , Y. Hu , Green Chem. 2021, 23, 501.

[31] R. Dinu , A. Pidvorotnia , D. D. Swanson , A. Mija , Chem. Eng. J. 2024, 499, 156486.

[32] J. L. Dubois , Manufacture of Maleic anhydride from Renewable Materials, Maleic Anhydride Obtained, and Uses Thereof, US 2012/0015411 A1, 2012.

[33] J. Lan , Z. Chen , J. Lin , G. Yin , Green Chem. 2014, 16, 4351.

[34] X. Li , J. Ko , Y. Zhang , ChemSusChem 2018, 11, 612. 29243400 10.1002/cssc.201701866

[35] R. Cucciniello , D. Cespi , M. Riccardi , E. Neri , F. Passarini , F. M. Pulselli , Green Chem. 2023, 25, 5922.

[36] S. Thiyagarajan , H. C. Genuino , M. Śliwa , J. C. Van Der Waal , E. De Jong , J. Van Haveren , B. M. Weckhuysen , P. C. A. Bruijnincx , D. S. Van Es , ChemSusChem 2015, 8, 3052. 26235971 10.1002/cssc.201500511

[37] X. Gao , X. Tong , Y. Zhang , S. Xue , IScience 2023, 26, 107203. 37485350 10.1016/j.isci.2023.107203 PMC10362136

[38] H. E. Andrews , Phthalic Anhydride 1920, 1336, 182.

[39] E. Mahmoud , D. A. Watson , R. F. Lobo , Green Chem. 2014, 16, 167.

[40] S. Giarola , C. Romain , C. K. Williams , J. P. Hallett , N. Shah , Chem. Eng. Res. Des. 2016, 107, 181.

[41] L. Shang , X. Zhang , M. Zhang , L. Jin , L. Liu , L. Xiao , M. Li , Y. Ao , J. Mater. Sci. 2017, 53, 5402.

[42] M. S. McMaster , T. E. Yilmaz , A. Patel , A. Maiorana , I. Manas‐Zloczower , R. Gross , K. D. Singer , ACS Appl. Mater. Interfaces 2018, 10, 13924. 29620846 10.1021/acsami.7b19085

[43] W. Xie , S. Huang , D. Tang , S. Liu , J. Zhao , Chem. Eng. J. 2020, 394, 123667.

[44] W. Xie , S. Huang , S. Liu , J. Zhao , Chem. Eng. J. 2021, 404, 126598.

[45] A. Bolduc , L. Rivier , S. Dufresne , W. G. Skene , Mater. Chem. Phys. 2012, 132, 722.

[46] A. E. Bejan , M. D. Damaceanu , Synth. Met. 2020, 268, 116498.

[47] S. Ota , K. Michishio , M. Harada , Mater. Today Commun. 2022, 31, 103501.

[48] D. Gay , S. V. Hoa , S. W. Tsai , Composite Mater.‐Design App, 3rd ed., CRC Press Taylor & Francis Group, Boca Raton 2015.

[49] M. Biron , Thermosets and Composites Material Selection, Applications, Manufacturing and Cost Analysis, 2nd Ed., Elsevier, Amsterdam 2014.

[50] Q. Guo , Thermosets – Structure, Properties, and Applications, 2nd Ed., Elsevier, Amsterdam 2017.

[51] I. M. Kalogeras , H. E. Hagg Lobland , J. Mater. Edu. 2012, 34, 69.

[52] M. Fache , A. Viola , R. Auvergne , B. Boutevin , S. Caillol , Eur. Polym. J. 2015, 68, 526.

[53] B. Dębska , L. Lichołai , Constr. Build. Mater. 2016, 124, 11.

[54] A. Nafees , G. Kalita , M. K. Paul , A. Sinha , N. V. S. Rao , RSC Adv. 2015, 5, 7001.

[55] R. H. Contreras , C. F. Tormena , L. C. Ducati , T. Llorente , Chem. Shift Trend. Light Atoms 2013, 3, 315.

[56] C. Li , A. Strachan , Polymer (Guildf) 2016, 97, 456.

[57] S. Liu , C. K. Schauer , J. Chem. Phys. 2015, 142, 4907365. 10.1063/1.4907365 25662636

[58] V. D. Mai , S. R. Shin , D. S. Lee , I. Kang , Polymers (Basel) 2019, 11, 293. 30960277 10.3390/polym11020293 PMC6419216

[59] J. Ma , G. Li , X. Hua , N. Liu , Z. Liu , F. Zhang , L. Yu , X. Chen , L. Shang , Y. Ao , Polym. Degrad. Stab. 2022, 201, 109989.

[60] S. Tripathi , H. Supriya , S. Bose , SPE Polymers 2024, 5, 136.

[61] D. W. van Krevelen , Polymer (Guildf) 1975, 16, 615.

[62] A. Kumar , J. T'Ien , Int. J. Spray Combustion Dynam.. 2012, 4, 299.

[63] M. Fei , Y. C. Chang , C. Hao , L. Shao , W. Liu , B. Zhao , J. Zhang , Compos B Eng. 2023, 248, 110366.

[64] H. Nabipour , X. Wang , Y. Hu , Eur. Polym. J. 2023, 194, 112166.

[65] H. Nabipour , H. Niu , X. Wang , S. Batool , Y. Hu , React. Funct. Polym. 2021, 168, 105034.

[66] A. A. C. Pereira , J. R. M. D'Almeida , Polímeros 2016, 26, 30.

[67] A. Toscano , G. Pitarresi , M. Scafidi , M. Di Filippo , G. Spadaro , S. Alessi , Polym. Degrad. Stab. 2016, 133, 255.

[68] G. Capiel , J. Uicich , D. Fasce , P. E. Montemartini , Polym. Degrad. Stab. 2018, 153, 165.

[69] P. J. Flory , Principles of Polymer Chemistry, Cornell University Press, Ithaca, New York, 1953.

[70] A. V. Tobolsky , Properties and Structure of Polymers, Wiley, New York, 1960.

[71] ASTM‐D2765‐16, Determination of gel content and swell ratio of cross–linked ethylene plastics, 2016.

[72] ASTM D570‐982018, Water Absorption of Plastics, 2018, 10.1520/D0570-98R18.

