# Supplementary material for: Sustainable Development of High‐performance Poly(ester‐imine) Biobased Thermosets
Source: Adv Sci (Weinh). 2025 Apr 25;12(26):2503483. doi: 10.1002/advs.202503483 (PMC12245105; doi:10.1002/advs.202503483)
Supplement: Supplementary file 1 — Supporting Information [file ADVS-12-2503483-s001.docx]

Supporting Information

Sustainable development of high-performance poly(ester-imine) biobased thermosets

Roxana Dinu^1^, Sandu Cibotaru^1^, David D. Swanson^2^ and Alice Mija^1^*

**List of Abbreviations**

| Acronym | Name |
| --- | --- |
| GA | **Glutaric Anhydride** |
| IA | **Itaconic Anhydride** |
| MA | **Maleic Anhydride** |
| SA | **Succinic Anhydride** |
| CA | **Citraconic Anhydride** |
| MNA | **Methyl Nadic Anhydride** |
| HHPA | **Hexahydro Phthalic Anhydride** |
| THPA | **cis-1,2,3,6- Tetrahydrophthalic Anhydride** |
| DDSA | **Dodecenylsuccinic Anhydride** |
| HMPA | **Hexahydro-4-Methyl Phthalic Anhydride** |
| EP-VAN-AP | **4-((oxiran-2-yl)methoxy)-N-(4-((oxiran-2-yl)methoxy)-3-methoxybenzylidene)benzenamine** |
| EP-VAN-DiAP | **N1,N4-bis(4-((oxiran-2-yl)methoxy)-3-methoxybenzylidene)benzene-1,4-diamine** |
| EP-SYR-AP | **4-((oxiran-2-yl)methoxy)-N-(4-((oxiran-2-yl)methoxy)-3,5-dimethoxybenzylidene)benzenamine** |
| EP-SYR-DiAP | **N1,N4-bis(4-((oxiran-2-yl)methoxy)-3,5-dimethoxybenzylidene)benzene-1,4-diamine** |

**1. Differential Scanning Calorimetry (DSC)**

**Figure S1.** DSC thermogram on the heating at 10 °C/min the EP-VAN-AP (black line), EP-VAN-DIAP (red line), EP-SYR-AP (blue line), and EP-SYR-DiAP (green line) Schiff based epoxy monomers

**2. Attenuated Total Reflection Fourier Transform Infrared Spectroscopy (ATR-FTIR)**

**Figure S2.** Dynamic temperature-dependent FT-IR spectra of EP-VAN-AP/ MA system from 40 to 250 °C in the region a) 3400‒2800 cm^-1^; b)1900‒1320 cm^-1^; c) 1320‒930 cm^-1^, d) 930‒550 cm^-1^


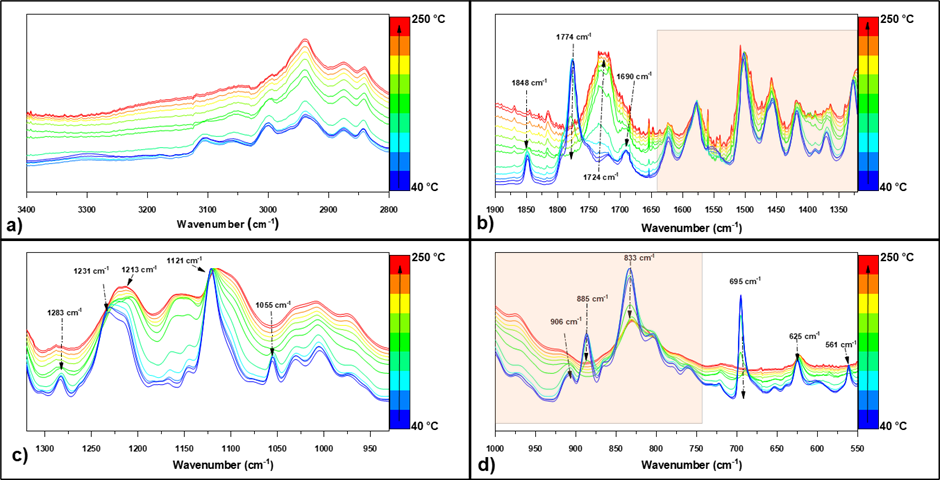


**Figure S3.** Dynamic temperature-dependent FT-IR spectra of EP-SYR-DiAP/ MA system from 40 to 250 °C in the region a) 3400‒2800 cm^-1^; b)1900‒1320 cm^-1^; c) 1320‒930 cm^-1^, d) 930‒550 cm^-1^


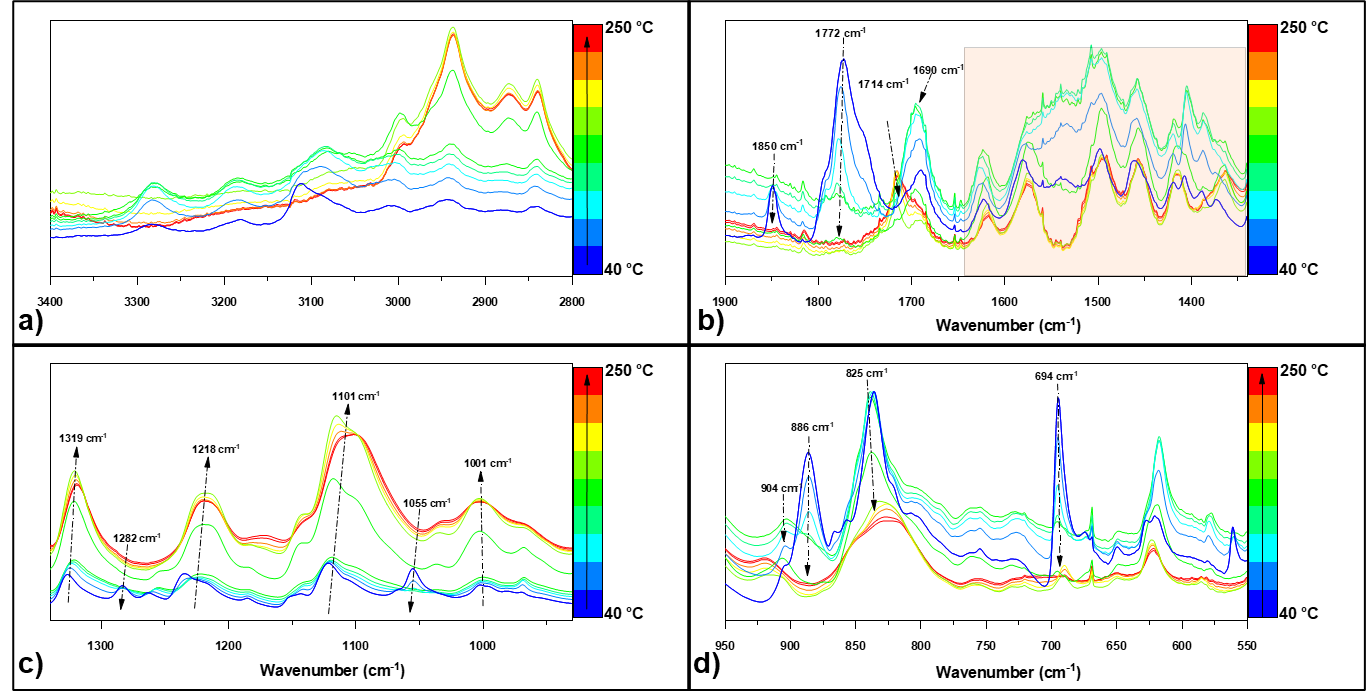


**3. Dynamic Mechanical Analysis (DMA)**

**Figure S4.** Loss moduli (E’’) vs temperature curves of the a) EP-VAN-AP, b) EP-VAN-DiAP, c) EP-SYR-AP, and d) EP-SYR-DiAP based thermosets developed with various anhydrides


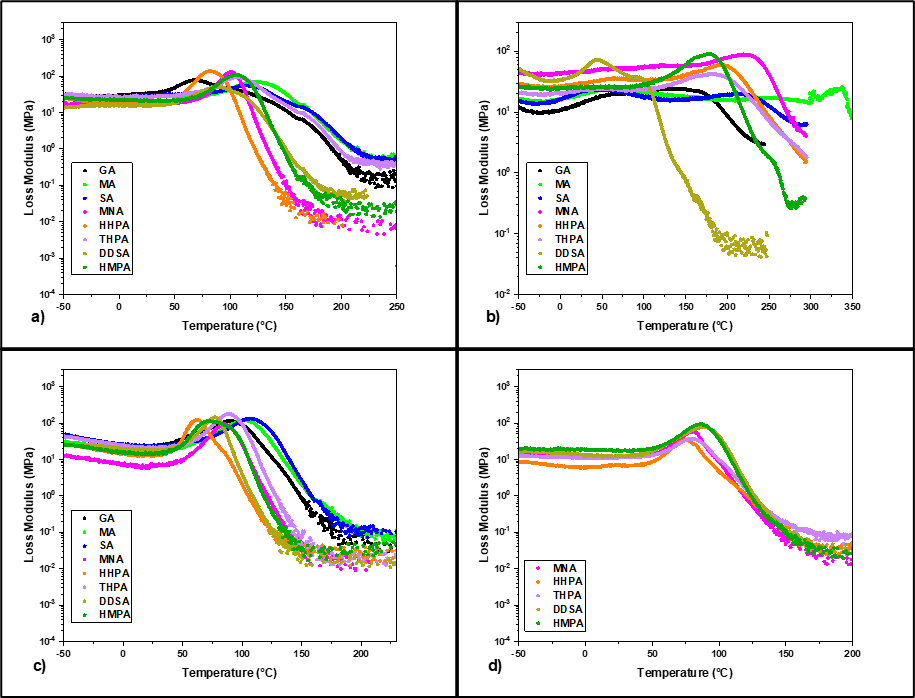


**Table S1.** Data comparison of the actual thermosets with published ones

| **Schiff-base** | **Curing agent** | **T_g_ [°C]** | **E’ [MPa]** | **LOI [%]** | **Ref.** |
| --- | --- | --- | --- | --- | --- |
|  EP-VAN-AP |  SA | 132 & 167 | 1670 | 28.9 | This study |
|  EP-VAN-DiAP |  MA | 334 | 650 |  |  |
|  EP-SYR-AP |  SA | 131 | 1670 | 31.4 |  |
|  EP-SYR-DiAP |  HHPA | 136 | 360 | 30 |  |
|  |  | 181 | ‒ | ‒ | [1] |
|  |  | 220 | 3602 | 35.5 | [2] |
|  |  | 117 | 2118 | ‒ | [3] |
|  |  | 197 | 2672 | 33.2 | [4] |
|  |  | 110 | 2672 | 34 | [5] |
|  |  | 150 | ‒ | 35.1 | [6] |
|  |  | 224 | 3383 | 38.5 | [7] |
|  |  | 200 | 2299 | 34.1 | [8] |
|  |  | 147 | ‒ | 39.5 | [9] |
|  |  | 204 | 2188 | 40 | [10] |
|  |  | 172 | 2112 | ‒ | [11] |
|  |  | 170 | 3721 | 32 | [12] |

**4. Thermogravimetric analysis**

**Figure S5.** TGA and DTG curves vs temperature of the a) EP-VAN-AP, b) EP-VAN-DiAP,c) EP-SYR-AP, and d) EP-SYR-DiAP based thermosets developed with various anhydrides in inert atmosphere, heating at 10 °C/min


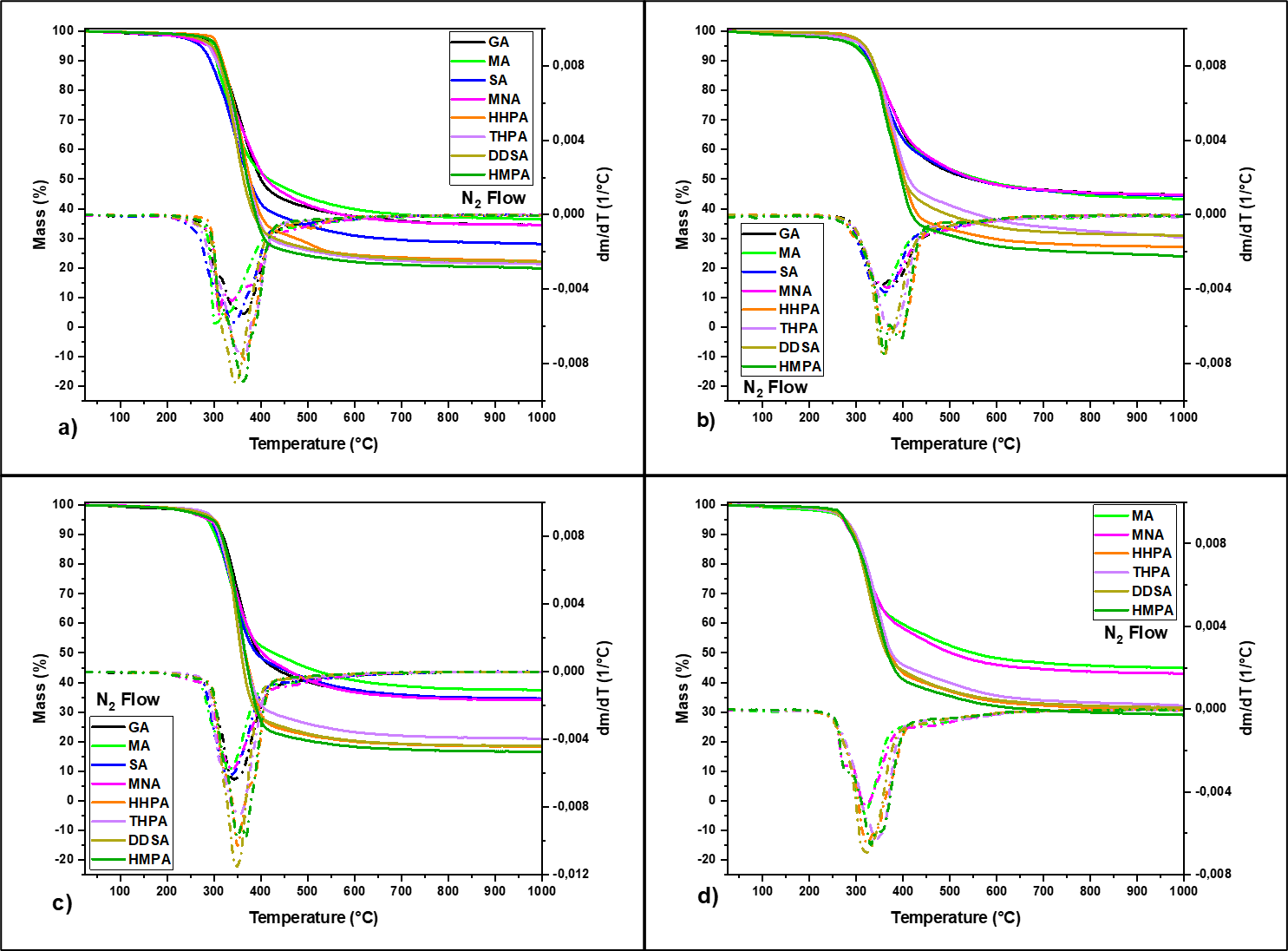


**5. Water Contact Angle**

**Figure S6.** Water contact angle of the a) EP-VAN-AP, b) EP-VAN-DiAP, c) EP-SYR-AP, and d) EP-SYR-DiAP Schiff based thermosets developed with various anhydrides


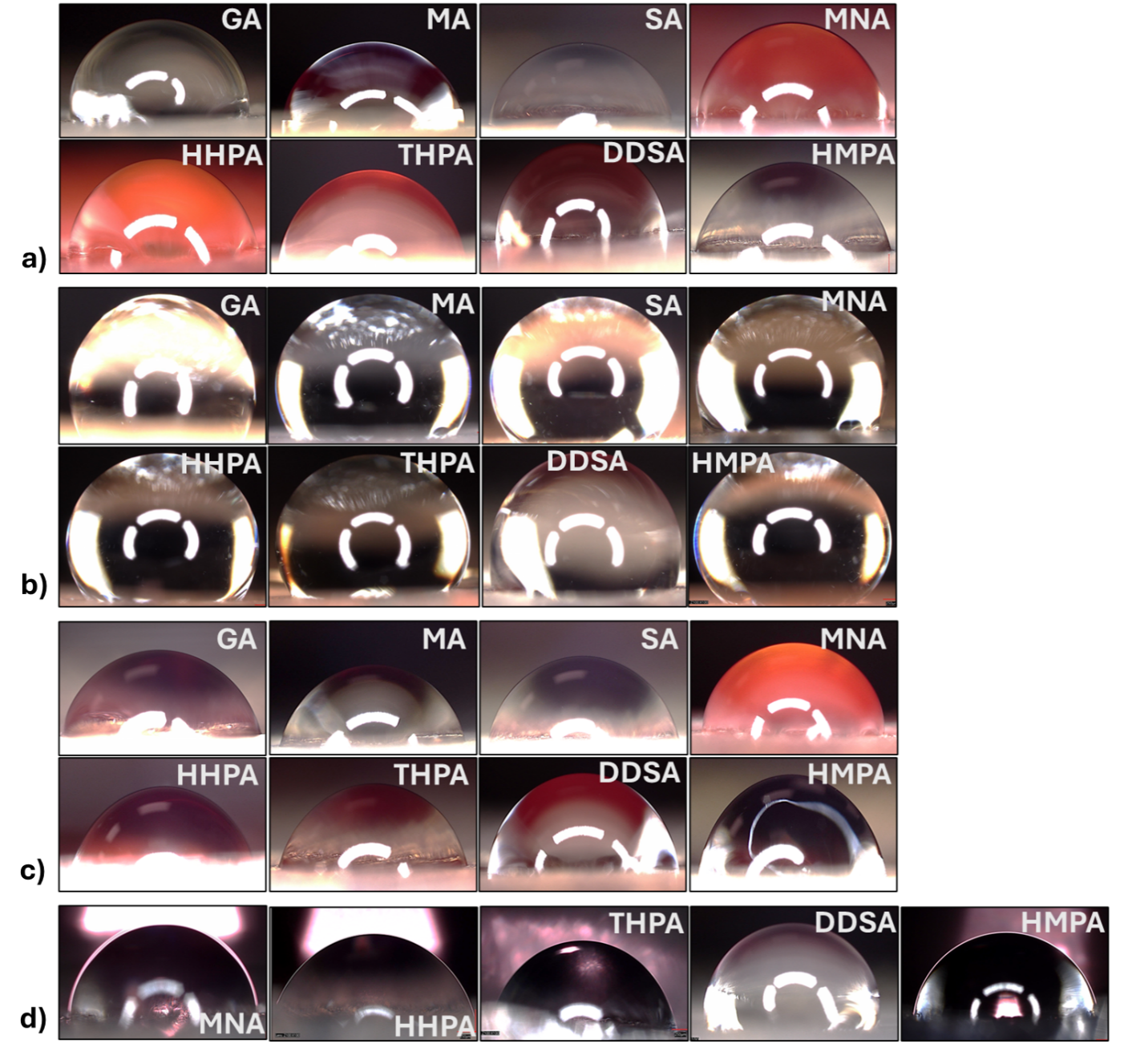


**References Supporting Info**

[1] X. Xu, S. Ma, J. Wu, J. Yang, B. Wang, S. Wang, Q. Li, J. Feng, S. You, J. Zhu, High-performance, command-degradable, antibacterial Schiff base epoxy thermosets: synthesis and properties, J. Mater. Chem. A. 7 (2019) 15420–15431. https://doi.org/10.1039/C9TA05293C.

[2] J. Li, Z. Weng, Q. Cao, Y. Qi, B. Lu, S. Zhang, J. Wang, X. Jian, Synthesis of an aromatic amine derived from biomass and its use as a feedstock for versatile epoxy thermoset, Chem. Eng. J. 433 (2022) 134512. https://doi.org/10.1016/j.cej.2022.134512.

[3] E. Desnoes, L. Toubal, A.H. Bouazza, D. Montplaisir, Biosourced vanillin Schiff base platform monomers as substitutes for DGEBA in thermoset epoxy, Polym. Eng. Sci. 60 (2020) 2593–2605. https://doi.org/10.1002/pen.25497.

[4] C. Yang, X. Xia, Y. Xiao, G. Wei, W. Li, Y. Lu, Degradable, intrinsically flame-retardant, low-water-absorbing vanillin-derived epoxy thermoset with a Schiff base structure, Polym. Degrad. Stab. 221 (2024) 110666. https://doi.org/10.1016/j.polymdegradstab.2024.110666.

[5] J. Ma, G. Li, X. Hua, N. Liu, Z. Liu, F. Zhang, L. Yu, X. Chen, L. Shang, Y. Ao, Biodegradable epoxy resin from vanillin with excellent flame-retardant and outstanding mechanical properties, Polym. Degrad. Stab. 201 (2022) 109989. https://doi.org/10.1016/j.polymdegradstab.2022.109989.

[6] N. Xu, B. Wang, Z. An, Y. Liu, L. Liu, Z. Hu, Y. Huang, γ-Ray-sensitive motif-driven versatile epoxy thermoset: Highly efficient radiation degradation, intrinsic flame resistance and sustainable synthesis, Chem. Eng. J. 450 (2022) 138151. https://doi.org/10.1016/j.cej.2022.138151.

[7] H. Nabipour, S. Rohani, Y. Hu, A bio-based epoxy resin derived from syringaldehyde with excellent mechanical properties, flame retardant and high glass transition temperature, Polym. Degrad. Stab. 214 (2023) 110410. https://doi.org/10.1016/j.polymdegradstab.2023.110410.

[8] Z. Miao, C. Peng, L. Xia, H. Xu, S. He, C. Chi, J. Zhong, S. Wang, W. Luo, G. Chen, B. Zeng, L. Dai, Fire-Safe Fully Bio-Based Schiff Base Epoxy Thermosets with Excellent Mechanical Properties and Adjustable Degradability, ACS Appl. Polym. Mater. 5 (2023) 6325–6337. https://doi.org/10.1021/acsapm.3c00953.

[9] H. Niu, H. Nabipour, X. Wang, L. Song, Y. Hu, Phosphorus-Free Vanillin-Derived Intrinsically Flame-Retardant Epoxy Thermoset with Extremely Low Heat Release Rate and Smoke Emission, ACS Sustain. Chem. Eng. 9 (2021) 5268–5277. https://doi.org/10.1021/acssuschemeng.0c08302.

[10] H. Nabipour, X. Wang, L. Song, Y. Hu, A high performance fully bio-based epoxy thermoset from a syringaldehyde-derived epoxy monomer cured by furan-derived amine, Green Chem. 23 (2021) 501–510. https://doi.org/10.1039/D0GC03451G.

[11] S. Wang, S. Ma, Q. Li, X. Xu, B. Wang, W. Yuan, S. Zhou, S. You, J. Zhu, Facile in situ preparation of high-performance epoxy vitrimer from renewable resources and its application in nondestructive recyclable carbon fiber composite, Green Chem. 21 (2019) 1484–1497. https://doi.org/10.1039/C8GC03477J.

[12] H. Nabipour, H. Niu, X. Wang, S. Batool, Y. Hu, Fully bio-based epoxy resin derived from vanillin with flame retardancy and degradability, React. Funct. Polym. 168 (2021) 105034. https://doi.org/10.1016/j.reactfunctpolym.2021.105034.
